# Soil to Synapse: Molecular Insights into the Neurotoxicity of Common Gardening Chemicals in Alzheimer’s and Parkinson’s Disease

**DOI:** 10.3390/ijms26136468

**Published:** 2025-07-04

**Authors:** Niti Sharma, Seong Soo A. An

**Affiliations:** Department of Bionano Technology, Gachon Bionano Research Institute, Gachon University, 1342 Seongnam-daero, Sujung-gu, Seongnam-si 461-701, Gyeonggi-do, Republic of Korea; nitisharma@gachon.ac.kr

**Keywords:** gardening chemicals, herbicides, mode of action, neurodegeneration, Alzheimer’s disease, Parkinson’s disease, gut–brain axis

## Abstract

The common gardening herbicides and fertilizers are crucial for weed control and plant growth, yet they may have potentially harmful impacts on neurological health. This review explored the possible effects of these chemicals on neurodegenerative disorders, especially Alzheimer’s disease (AD) and Parkinson’s disease (PD). The mode of action of several frequently used gardening chemicals (paraquat, glyphosate, 2,4-dichlorophenoxyacetic acid: 2,4-D, and ammonium chloride) in AD and PD has been highlighted. The mechanisms involved are glutamate excitotoxicity, dopaminergic pathway disruption, oxidative stress, mitochondrial dysfunction, neuroinflammation, synaptic dysfunction, and gut–brain-axis dysregulation, crucial in the pathophysiology of AD and PD. Although the links between these substances and neurodegenerative conditions remained to be seen, growing evidence indicated their detrimental effects on brain health. This highlights the need for further research to understand their long-term consequences and develop effective interventions to mitigate the adverse effects of commonly used chemicals on human health and the environment.

## 1. Introduction

Neurodegenerative diseases (NDs) are characterized by progressive synaptic failure, neural network disruptions, and the accumulation of physicochemically modified protein variants within the brain. NDs impact millions of individuals across the globe. Alzheimer’s disease (AD) is one of the most prevalent ND, and according to estimates, there are 32 million people with AD dementia, 69 million with prodromal AD, and 315 million with preclinical AD worldwide [1]. Parkinson’s disease (PD) is the second most common ND worldwide, with a prevalence of approximately 10 million affected people globally [2]. While age and genetics remained the primary risk factors for the onset of all NDs, environmental stimuli (pollutants, toxins, and heavy metals) would significantly raise the risk for NDs.

Fertilizers, pesticides, and herbicides are regularly employed in residential gardens to improve soil nutrients and manage pests and undesirable vegetation. Typical examples consisted of paraquat (PQ), glyphosate, 2,4-dichlorophenoxyacetic acid (2,4-D) (a component of Agent Orange), and ammonium chloride. China is the largest manufacturer of PQ, producing over 100,000 tons per year [3]. Glyphosate is the most commonly used herbicide, with 740,000 to 920,000 tons of use predicted for the year 2025 [4]. It is used frequently as it is less toxic than the others, displaying a lethal dose (LD_50_) of 4230 mg/kg/body weight (BW) than ammonium chloride (LD_50_: 1650 mg/kg/BW), 2,4,5-T (LD_50_: 390 mg/kg/BW), 2,4-D (LD_50_: 375 mg/kg/BW, debarred in 3 countries), and PQ (LD_50_:150 mg/kg/BW, debarred in 46 countries) [5,6]. The dioxin contaminant in Agent Orange (herbicide and defoliant used in the Vietnam War) is most toxic with an LD_50_ of 107 μg/kg when administered subcutaneously in humans [7]. Despite a ban on its use in 1971, an analogous blend is still being used by US forest departments across the country [8]. If not managed appropriately, these chemicals can be harmful to humans and may pollute soil and water resources.

The human body can be exposed to common herbicides like PQ, glyphosate, 2,4-D, and similar substances through ingestion, inhalation, or skin absorption [9]. They enter the body and may reach the brain either through the bloodstream by crossing the blood–brain barrier (BBB) or indirectly by triggering systemic inflammation and oxidative stress [10]. Research indicated that exposure to pesticides had detrimental impacts, linked to reproductive and developmental toxicity [11,12], besides raising the likelihood of developing respiratory disorders, cancer, and NDs [13,14,15,16]. Mostly, these chemicals manifested their detrimental effects through oxidative stress, bringing DNA damage, lipid peroxidation, inflammation, and immune evasion, which could subsequently lead to various ailments [17,18]. Notably, there is mounting evidence that early pathological alterations in NDs like AD and PD may have their origins outside of the brain, specifically in the olfactory or gastrointestinal systems. According to the widely discussed “dual-hit” hypothesis, harmful substances may first affect the gastrointestinal tract or the olfactory system, and then spread to the brain through the vagus nerve or olfactory pathways [19,20]. Early accumulation of α-synuclein in the gut and olfactory bulb in PD, and amyloid-β in the olfactory system in AD, supports this concept [21]. However, evidence from neuropathological studies suggests that not all PD align with the dual-hit hypothesis [22]. Notably, a subset of individuals does not exhibit α-synuclein pathology in the dorsal motor nucleus of the vagus (DMV), a key region implicated in the gut-to-brain transmission route, indicating that alternative initiation sites may exist in PD pathogenesis [23]. While the exact entry route may vary depending on the chemical and individual factors, these peripheral pathways likely play a key role in how environmental exposures contribute to neurodegeneration.

The gut microbiota is now recognized to have a crucial and increasingly better-defined role in the pathogenesis of AD and PD through what’s termed the gut–brain axis—a two-way bidirectional communication network between the gut and CNS. This communication is mediated by various factors, including chemicals produced by the gut microbiome and environmental contaminants [24,25,26]. Pesticide exposure can alter the balance and diversity of the gut microbiota, leading to dysbiosis. Recently, Chen et al. [27] studied the metabolic alterations in the gut microbiota caused by several pesticides (organochlorides, organophosphates, and pyrethroids). The study showed that even minute amounts (0.05–1 μg/mL) of these chemicals can either boost or block the growth of gut bacteria. The bioaccumulation of pesticides in the gut potentially increases health risks by disrupting hundreds of key metabolic processes, including those related to amino acids, sugars, and fats [27].

In this work, the molecular mechanisms involved in the neurotoxic actions of some commonly used gardening chemicals were reviewed with a particular emphasis on the mechanisms associated with neurodegenerations in AD and PD.

## 2. Materials and Methods

This review aimed to comprehensively analyze existing research articles related to common gardening chemicals in neurodegeneration. The literature search was carried out in the specialized databases PubMed, Google Scholar, and Web of Science for relevant and important information until May 2025. The search terms used were “paraquat”, “glyphosate”, “ammonium chloride”, “Agent Orange”, “2,4-dichloro phenoxy acetic acid”, “2,4,5-Trichlorophenoxyacetic acid”, with the filters “Alzheimer’s disease”, “Parkinson’s disease”, “neurodegeneration”, and “gut–brain-axis”. Exclusion criteria: articles not specifying neurodegeneration were excluded. Non-English literature was excluded.

## 3. Mode of Action of Some Common Gardening Chemicals in Neurodegeneration

**Figure 1:**
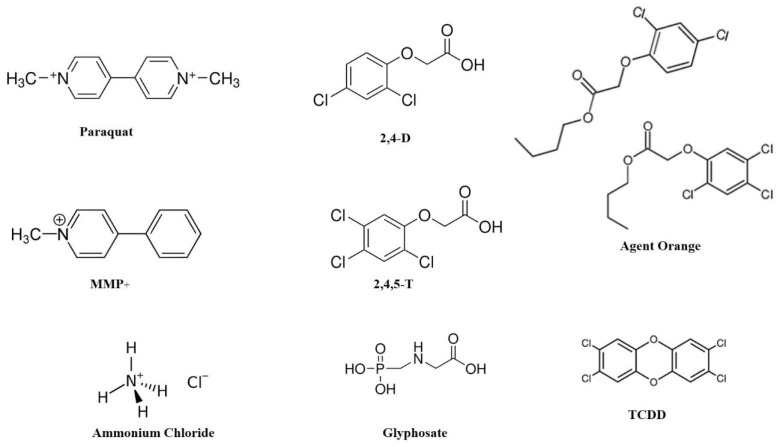
Chemical structures of some common gardening chemicals. MMP^+^ is a neurotoxin with a structural resemblance to paraquat. Agent Orange is a 1:1 cocktail of 2,4-D and 2,4,5-T. TCDD is a contaminant present in Agent Orange. Abbreviations: 2,4-D: 2,4-dichloro phenoxy acetic acid; 2,4,5-T: 2,4,5 trichloro phenoxy acetic acid; MMP^+^: 1-methyl-4-phenyl pyridinium; TCDD: 2,3,7,8-Tetrachlorodibenzodioxin.

Herbicides are commonly used in farming and gardening to control weeds, but increasing evidence points to their possible neurotoxic effects; hence, their contribution to neurodegenerative conditions has come under scrutiny. The most prevalent herbicides, including PQ, glyphosate, 2,4-D, and ammonium chloride (Figure 1), have been linked with neurotoxicity by different mechanisms.

PQ is an extremely toxic bipyridinium herbicide with a structural resemblance to 1-methyl-4-phenylpyridinium (MPP^+^), an infamous neurotoxin (Figure 1). Both PQ and MMP^+^ have been implicated in inducing PD, albeit through distinct mechanisms [28]. MPP^+^, a toxic metabolite of N-methyl-4-phenyl-1,2,3,6-tetrahydropyridine (MPTP), is transported into the dopaminergic neurons through dopamine transporter (DAT), where it inhibits mitochondrial complex I, leading to neurotoxicity. In contrast, PQ is largely transported via organic cation transporter-3 (OCT3) and does not inhibit mitochondrial complex I. Instead, it induces oxidative damage by generating free radicals and lipid peroxidation in the brain [28]. Glyphosate, the active ingredient in most commercial herbicides, is also associated with neuroinflammation and disruption of the gut–brain axis, the factors contributing to the pathogenesis of both AD and PD [29]. Equivalently, 2,4-D, an active ingredient of the infamous Agent Orange, was found to induce oxidative stress, neuroinflammation, and dopaminergic neurodegeneration [30]. Ammonium chloride (NH_4_Cl), though less studied, is speculated to impact neuronal homeostasis by acid-base balance disturbances and cellular metabolic mechanisms. In addition, it disrupts the homeostasis of chloride ions (Cl^−^) within and outside neurons essential factor for proper GABAergic inhibitory signaling. Disturbance of chloride ion balance may hinder GABAergic inhibition, potentially leading to excessive neuronal excitation. Nonetheless, overexcitation can also arise from various factors, including increased glutamatergic signaling, modified ion channel activity, and neuroinflammatory reactions [31]. As these chemicals were employed extensively and internationally, investigating their possible connections with neurodegenerations is important for public health, policy-making, and identifying safer alternatives. The detailed mode of action of these chemicals has been discussed in the following sections.

### 3.1. Paraquat (PQ)

PQ (1,1′-dimethyl-4,4′-bipyridylium dichloride or methyl viologen; [(C_6_H_7_N)_2_] Cl_2_) is a widely used contact herbicide. It is a quick-acting and non-selective chemical that kills leaves and stems, but not the roots, as it does not travel through the plant’s vascular system. It targets the electron transfer chain (ETC) in the photosystem I (PS-I) to generate the toxic reactive oxygen species (ROS), eventually killing the plant [32]. While PQ is strongly attached to the soil particles, it can quickly depreciate upon light exposure and can stay active and transportable in water. PQ is toxic to humans and can be absorbed through the skin, digestive tract [33], and placental barrier [34], causing multiple organ failure. It is not metabolically transformed in the body and is excreted unchanged through the kidneys [35]. Approximately 20% of the ingested PQ is absorbed in the stomach, after which it primarily distributes to the lungs, kidneys, liver, and muscles. PQ is metabolized by redox cycling catalyzed by several enzymes: NADPH-cytochrome P_450_ reductase (CPR), xanthine oxidase (XO), and nitric oxide synthase (NOS), producing ROS and reactive nitrogen species (RNS). This cycle oxidizes lipids and proteins and damages DNA, especially in neurons, astrocytes, and endothelial cells [36].

#### 3.1.1. BBB Permeability

The capacity of PQ to penetrate the BBB is moderately intricate. The response hinged on the manner and location of its crossing. It could cross through limited passive diffusion, as observed in a study where only 0.05% of injected PQ reached the brain within the first hour and remained in the cerebro-circulatory system after 24 h [37]. The results suggested that PQ does not easily penetrate the BBB, but could accumulate in areas of the brain, lacking a BBB, including the pineal gland, cerebral ventricular linings, olfactory bulb, and hypothalamus [37]. PQ’s transport across the BBB is possible in conditions when the brain is damaged due to any reason (NDs, aging, inflammation) [10,38]. Research indicated that after multiple low-dose exposures, PQ can penetrate the BBB by increasing ROS generation in the brain [39]. Microglia play a significant role in PQ transport through their connections with the BBB. PQ exposure triggers microglia by activating NADPH oxidase (NOX2), elevating ROS, leading to increased BBB permeability by damaging the epithelial cells [38]. In addition, unwarranted activation of the phosphatidylinositol 3-kinase/protein kinase B (PI3K/AKT) pathway may augment interleukin 6 (IL-6) production, oxidative stress, and inflammation. Thus, persistent exposure to PQ affects BBB functionality and injures the brain tissue.

The other routes of PQ transport mainly include OCT3, which is present in the endothelial cells of the BBB, astrocytes, and neurons. OCT3 plays a major role in mediating PQ uptake in glial and neuronal cells and is considered the most significant transporter for its neurotoxicity [40]. In contrast, L-type amino acid transporter 1 (LAT1), which is highly expressed at the BBB, has the potential to facilitate PQ’s entry into the brain, but it is not the primary route. Additionally, the DAT (Na^+^/Cl^−^-dependent symporter) expressed on dopaminergic neurons, selectively transports monovalent cationic substrates like PQ^+^ (closer physicochemical resemblance to MPP^+^) rather than the usual divalent cation (PQ^2+^), which is not a suitable substrate for it. In biological systems, the occurrence and stability of PQ^+^ are minimal, which is why its involvement in PQ neurotoxicity through DAT is uncertain [41].

#### 3.1.2. Mechanism of Demyelination

Myelin is a lipid-rich sheath covering the nerve cell axons to insulate them and enhance the speed at which electrical signals (action potentials) travel along the axon. Insulation is crucial for effective motor, sensory, and cognitive functions. Demyelination leads to reduced nerve conduction and degeneration of neurons, all of which contribute to cognitive decline and motor impairments, the key characteristics of neurodegenerative conditions [42,43]. PQ leads to demyelination mainly by inducing oxidative stress and neuroinflammation [44]. PQ also downregulates the expression of genes involved in myelin integrity (Claudin 5:Cldn5), myelin maintenance (UDP galactosyltransferase 8A: Ugt8a and SRY-box-containing gene: Sox10), oligodendroglia proliferation and differentiation (Sox2); Schwann cell migration and myelination (human epidermal growth factor receptor 3: HER3) [45].

The oxidative stress generated by PQ results in lipid peroxidation of the myelin sheath, causing structural and functional damage [46]. Microglial activation by increased ROS releases pro-inflammatory cytokines that damage oligodendrocytes, the cells responsible for myelin production and maintenance. The resulting BBB disruption allows peripheral immune cells to infiltrate the central nervous system (CNS), which enhances immune-mediated demyelination [44]. In addition, PQ hinders the removal of glutamate by astrocytes, resulting in an excessive buildup of glutamate and excessive activation of N-methyl-d-aspartate receptors (NMDAR), which raises intracellular calcium levels, resulting in oligodendrocyte apoptosis [43].

#### 3.1.3. Mechanism of Neurodegeneration in AD

PQ has been shown to induce oxidative stress, particularly within the mitochondria of neurons, which increases Aβ levels in the brain [47] and activates NLR family pyrin domain containing 3 (NLRP3) inflammasome [48]. PQ-induced oxidative stress inhibited neural progenitor cells (NPCs) and affected the wingless-type protein (Wnt) pathway in mice [49]. In particular, PQ modulates Wnt via microRNA (miRNA) in human neural progenitor cells (hNPCs) with direct relevance to AD [50]. Wnt signaling is recognized to be dysfunctional in AD, and stimulation of the Wnt/β-catenin signaling pathway inhibits β-secretase (BACE-1) expression, inhibits Aβ production, and tau hyperphosphorylation in the brain [51].

PQ has also been known to inhibit hippocampal neurogenesis, interfere with hippocampus-dependent learning and memory, and reduce AKT phosphorylation [52]. AKT normally activates the mammalian target of rapamycin (mTOR), which is essential for cell growth and neurogenesis. Studies indicate that the activation of AKT could safeguard against neuronal cell loss in AD by inhibiting glycogen synthase kinase-3 beta (GSK-3β), a protein suppressing neurogenesis [53].

#### 3.1.4. Mechanism of Neurodegeneration in PD

Long-term repeated exposure to PQ can selectively impair the nigrostriatal dopaminergic (DA) system, affecting locomotion and cognitive abilities [54]. Prolonged PQ exposure induces oxidative stress, which eventually impairs the BBB and damages the brain tissue. This may be due to the overactivation of the PI3K/AKT signaling pathway, which leads to increased IL-6 production and oxidative stress, contributing to inflammation-related brain injuries [55]. PQ induced PD-like lesions in certain rodent models, but there was no evidence of causing human brain lesions directly [56]. PQ oxidizes the cytosolic thioredoxin 1 (TRX1) and activates c-Jun N-terminal kinase (JNK) and caspase-3, resulting in the apoptotic death of DA neurons. PQ might activate autophagy in neurons through apoptotic signal-regulating kinase 1 (ASK1) [57], a member of the mitogen-activated protein kinase family. In substantia nigra (SNr), PQ increased α-synuclein production and aggregation in a dose-dependent manner [58]. By activating NMDAR, PQ contributes to excitotoxicity and increased ROS. In turn, increased ROS worsens the situation by inhibiting glutamine synthase in astrocytes, reducing glutamate uptake, and finally causing neuronal cell death [59]. PQ increased the level of NADPH oxidase 1 (NOX1), which has been implicated in producing toxic chemicals called superoxide (O^2•−^), in rat brain cells (N27 cells). It also activated Ras-related C3 botulinum toxin substrate 1 (Rac1), a protein that is essential for NOX1 to produce these reactive species and trigger cellular damage [60]. PQ harms the brain by over-activating microglia through a specific pathway involving macrophage antigen complex-1 (Mac1), which triggers inflammation via NOX and NLRP3 inflammasome. This excessive inflammation leads to brain cell damage and memory problems [61]. Chronic exposure to PQ increased the levels of GSK-3β, a protein involved in several cellular functions and signaling, in the midbrain and pons but reduced those in the striatum [62]. PQ also damages key brain areas that control movement and reward, reducing the number of DA neurons in the brain [63]. In addition, the PQ-exposed rats also had decreased dopamine breakdown products (DOPAC) and noradrenaline in the striatum important for brain function, resulting in movement problems in the rats [64]. Thus, in short, besides promoting α-synuclein expression and aggregation, PQ influences several other pathogenic pathways, including oxidative stress, mitochondrial dysfunction, excitotoxicity via NMDA receptor activation, neuroinflammation (NOX1, Mac-1/NLRP3 activation), and apoptotic signaling (JNK/caspase-3 and ASK1 pathways).

Several epidemiological studies, such as the Agricultural Health Study (AHS), have indicated a higher risk of PD in people with job-related exposure to PQ. Tanner et al. (2011) found that individuals who used PQ faced about a 2.5-fold increased risk of developing PD (Odds ratio (OR): 2.5, 95% CI: 1.4–4.7) in contrast to non-users [65]. Likewise, a meta-analysis of data from cohort and case–control studies demonstrated that exposure to PQ was linked to a 2-fold increased risk for PD (strata odds ratio interval (sOR): 2.19; 95% CI: 1.48–3.26) [66]. A subsequent meta-analysis showed an association between PD and PQ exposure (OR 1.64; 95% CI: 1.27–2.13; I^2^ 24.8%) [67]. There is a statistically significant association between paraquat exposure and PD. Vaccari et al. (2019), on the other hand, note that the odds of PD given PQ exposure from the only cohort study were a non-significant 1.08 (95% CI: 0.57–2.04) and 1.25 (95% CI: 1.01–1.51) from case–control studies [68]. A critical review of reviews was undertaken, focusing on reviews published between 2006 and 2021, concluded that the relation between PD and PQ exposure is not confirmed [69]. Recently, Paul et al. showed that people who lived or worked near areas where PQ was regularly applied had a higher risk of developing PD (OR: 2.08, 95% 1.31, 3.38). Similar increased risks were also seen for people living near PQ-treated areas (OR: 1.91, 95% CI: 1.30–2.83). The strongest associations were found in individuals diagnosed with PD before age 60 [70].

**Figure 2:**
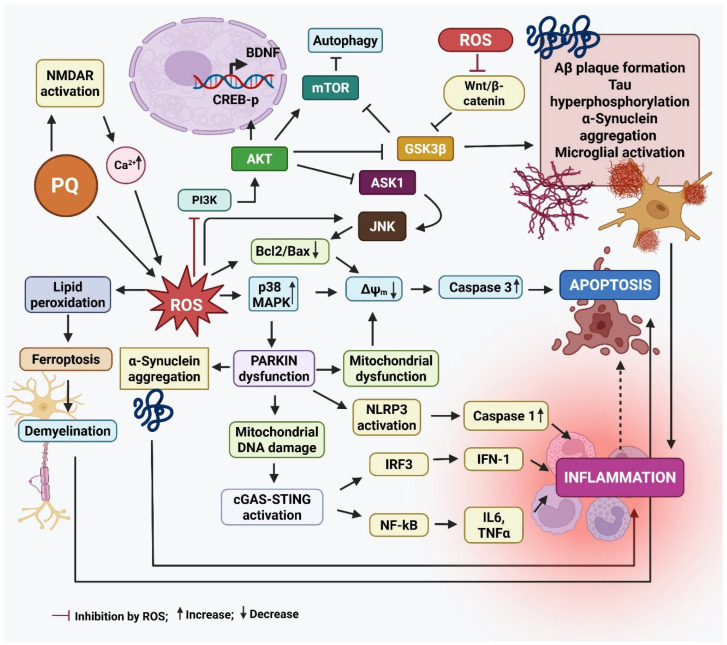
The mode of action of paraquat that results in neurodegeneration. Paraquat (PQ) plays a significant role in the development of Parkinson’s disease (PD) by increasing the production of α-synuclein, a protein that tends to clump together and form Lewy bodies, a key feature of PD. These protein aggregates contribute to the death of dopamine-producing neurons in the substantia nigra. PQ also activates stress-related signaling pathways, like Jun N-terminal kinase (JNK) and mitogen-activated protein kinase (p38 MAPKs), which push neurons toward programmed cell death (apoptosis). At the same time, it triggers the nuclear factor kappa-light-chain-enhancer of activated B cells (NF-κB) pathway, leading to the release of inflammatory molecules like tumor necrosis factor-α (TNF-α) and interleukin-1 beta (IL-1β), which worsens inflammation in the brain. Another damaging effect of PQ is its interference with the cell’s ability to clear damaged mitochondria—a process known as mitophagy—by disrupting the PINK1/Parkin pathway. This leads to dysfunctional mitochondria building up in neurons. Additionally, PQ induces ferroptosis, a type of cell death linked to iron buildup and oxidative damage to cell membranes, particularly in dopamine neurons. When Parkin, a protein critical for cellular cleanup systems, is deficient or dysfunctional, it causes misfolded proteins like α-synuclein to accumulate. Parkin dysfunction also increases DNA damage and may worsen inflammation through several pathways, including the NLR family pyrin domain containing 3 (NLRP3) inflammasome activation, and the cyclic GMP-AMP synthase-stimulator of interferon genes (cGAS–STING) pathway, all of which drive the production of inflammatory cytokines (e.g., IL-6, TNF-α, IL-1β, IFN-I). This chain of events sets off a vicious cycle: mitochondrial damage leads to inflammation, which in turn damages more neurons and mitochondria, amplifying the disease process in PD. In AD, PQ promotes the clumping of amyloid-beta (Aβ) proteins by increasing oxidative stress (ROS), making it harder for the brain to clear Aβ and damaging neuron membranes. It activates enzymes like Glycogen synthase kinase-3 beta (GSK-3β), which increase the abnormal phosphorylation of tau proteins, leading to the formation of neurofibrillary tangles—another major hallmark of AD. It also reduces the activity of cAMP-response element binding protein (CREB) and levels of brain-derived growth factor (BDNF), both of which are essential for memory and the ability of neurons to adapt and form new connections (synaptic plasticity). Furthermore, it impairs the Wnt/β-catenin pathway, a key signaling route in brain development and repair. When this pathway is disrupted, it can lead to cell death and prevent neural progenitor cells (NPCs) from multiplying and forming new neurons. Created by Biorender.com.

Figure 2 summarizes the main events leading to neurodegeneration by PQ exposure.

### 3.2. Glyphosate

Glyphosate (N-(phosphonomethyl)glycine) is an organophosphorus compound with the trade name of Roundup. It is a common, non-selective, and broad-spectrum herbicide. It stops the plants from producing specific proteins essential for their growth by interfering with the shikimic acid pathway via competitively inhibiting enzyme 5-enolpyruvylshikimate-3-phosphate synthase (EPSPS) [71]. The resulting inhibition of EPSP decreases the production of aromatic amino acids (phenylalanine, tyrosine, and tryptophan) that are essential for protein synthesis and plant development. Although glyphosate is degraded by soil microorganisms, resulting in its low levels, frequent use can increase its accumulation in the soil, potentially intensifying the likelihood of groundwater contamination [72]. Humans can be exposed to glyphosate primarily through ingestion or inhalation, the cutaneous uptake is minor. Glyphosate does not undergo significant metabolism in mammals and only a small amount (<1%) is metabolized to aminomethyl phosphonic acid (AMPA). Glyphosate and AMPA do not exhibit any tendency of bioaccumulation [73] and are eliminated from the body within 24–48 h [74].

#### 3.2.1. BBB Permeability

In vitro studies on brain microvascular endothelial cells (BMECs) have shown that glyphosate and AMPA can enhance BBB permeability. Glyphosate crosses the BBB through passive transcellular diffusion [75] and active transport mediated by LAT1 and LAT2 [76]. Mechanistically, glyphosate downregulates key tight junction proteins, disrupting endothelial barrier integrity. This facilitates the infiltration of peripheral immune cells into the brain parenchyma, initiating neuroinflammatory processes [75]. Once inside the CNS, glyphosate and AMPA further exacerbate BBB dysfunction and stimulate inflammatory responses, including upregulation of tumor necrosis factor-α (TNF-α) [77]. Though current evidence suggests a sound mechanistic connection between glyphosate exposure and disruption of the BBB, additional in vivo research is required to measure BBB penetration and evaluate subsequent neurological effects.

#### 3.2.2. Mechanism of Neurodegeneration in AD

Glutamate excitotoxicity is a key pathological mechanism in AD, contributing to synaptic dysfunction, neuronal loss, and cognitive decline. Glyphosate has been reported to inhibit glutamate transporters, leading to glutamate activity in the synapse, increasing NMDAR overstimulation, mitochondrial dysfunction, and Ca^2+^-mediated neuronal damage [78,79,80]. The activation of extracellular signal-regulated kinases (ERK) and Ca^2+^/calmodulin-dependent protein kinase II (CaMKII) signaling pathways by glyphosate was also shown to enhance glyphosate-induced neurotoxicity [81]. In a related study, glyphosate-based herbicides (GBH) exposure increased the CSF levels of excitatory amino acids, suggesting that GBH may cause an imbalance in the downstream metabolism of arginine and excitatory amino acids, which may worsen GBH-induced neurotoxicity [82]. Glyphosate elevated TNFα expression and soluble Aβ and disrupted the brain’s transcriptome in a dose-dependent manner [77]. Even after a 6-month recovery period after exposure, the byproduct of glyphosate (AMPA) remained detectable in the brain. This persistent presence of AMPA, along with neuroinflammation and AD-like pathology (elevated insoluble Aβ_42_ and hyperphosphorylated tau), suggested a detrimental impact of glyphosate on brain health [83].

A recent epidemiological study evaluated human exposure to glyphosate and used an AD memory test to explore a possible relationship between glyphosate exposure and cognitive function. The findings showed a substantial inverse association between urine glyphosate levels and CERAD-WLT (Consortium to establish a registry for Alzheimer’s disease word list memory test) scores, pointing to a link between increased long-term glyphosate exposure and cognitive loss [84]. Also, glyphosate exposure in older adults was positively associated with impaired performance on the CERAD Delayed Recall (DR) test, with an odds ratio (OR) of 1.52 (95% CI: 1.01–2.30; *p*: 0.049) [85], providing new epidemiological evidence of the harmful effect of glyphosate on cognitive functions. A more recent study has reported a significant inverse relationship between urinary glyphosate levels and performance on memory recall tasks, including the CERAD Delayed Recall test, with OR of 1.52 (95% CI: 1.01–2.30; *p*: 0.049) [85], frailty prevalence (adjusted OR: 1.14, 95% CI: 1.04–1.25; quartiles *p*: 0.002), and all-cause mortality (adjusted hazard ratio HR: 1.19, 95% CI: 1.07–1.31; *p*: 0.005) [86] indicating a possible link between glyphosate exposure and cognitive impairment, frailty, and all-cause mortality in older adults.

#### 3.2.3. Mechanism of Neurodegeneration in PD

Given that glyphosate is used extensively worldwide, exposure to it is probably a potential environmental risk factor for PD. Glyphosate exposure during gestation and lactation caused glutamate excitotoxicity and oxidative stress in the rat model through NMDAR activation, cholinergic transmission impairment, astrocyte dysfunction, ERK1/2 overactivation, and reduced phosphorylation of nuclear factor kappa light-chain enhancer of activated B cells (NF-κB) [80]. Glyphosate worsened MPTP-induced dopaminergic neurotoxicity in adult mice’s striatum and SNr [87]. Several research findings linked glyphosate exposure to neurodegeneration in PD through inhibition of acetylcholinesterase (AChE) activity, mitochondrial dysfunction, neuroinflammation, apoptosis, and oxidative stress [88,89]. Glyphosate caused cell death in neuronal-differentiated pheochromocytoma (PC12) cells in addition to activating apoptotic pathways. The deactivation of Beclin-1 reduced both apoptosis and autophagy in glyphosate-treated cells, suggesting its involvement in the cell death mechanisms induced by glyphosate [90].

In some epidemiological cases, glyphosate-induced parkinsonism has been reported several years after acute and chronic exposure [91,92]. The glyphosate neurotoxicity resulted in loss of dopaminergic neurons, resulting in rigidity, slowness, and resting tremor with no impairment of short-term memory [92]. It is speculated that acute GBH poisoning or long-term exposure could result in the early onset of PD [93].

#### 3.2.4. Effect on Hippocampal Long-Term Potentiation (LTP) and Learning

Poor hippocampus long-term potentiation (LTP) and learning are associated with both AD and PD through distinct mechanisms. Aβ oligomers and tau disrupt synapses and LTP, which causes AD to directly target the hippocampus, resulting in severe learning and memory difficulties [94]. Although oxidative stress and neuroinflammation are present in both disorders that further disrupt synaptic function, AD causes direct hippocampal injury from the start, while PD-related LTP impairments arise after dopamine dysfunction, and alpha-synuclein (α-synuclein) accumulation further deteriorates the hippocampal function [95].

Recently, the effect of glyphosate on synaptic transmission and LTP induction was studied in vivo and ex vivo rat hippocampal slices. The herbicide reduced synaptic transmission in the hippocampal Cornu Ammonis 1 (CA1) area and had adverse effects on memory and learning. Nevertheless, the effect was reversed by pretreatment with TLR4 antagonist (TAK-242) and microglia inhibitor, minocycline [96], suggesting the involvement of neuroinflammation in this process. Astrocytic dysfunction, which is often initiated by microglia, can influence not only neuronal survival but also stimulate further microglial activation. Glyphosate stimulated mitochondrial respiratory chain enzymes in a rat astroglioma cell line [97] and elevated the expression of astrocyte marker, glial fibrillary acidic protein (GFAP) [98]. Therefore, alterations in astrocytic function brought on by this herbicide may be a factor in neuroinflammation and LTP [99].

**Figure 3:**
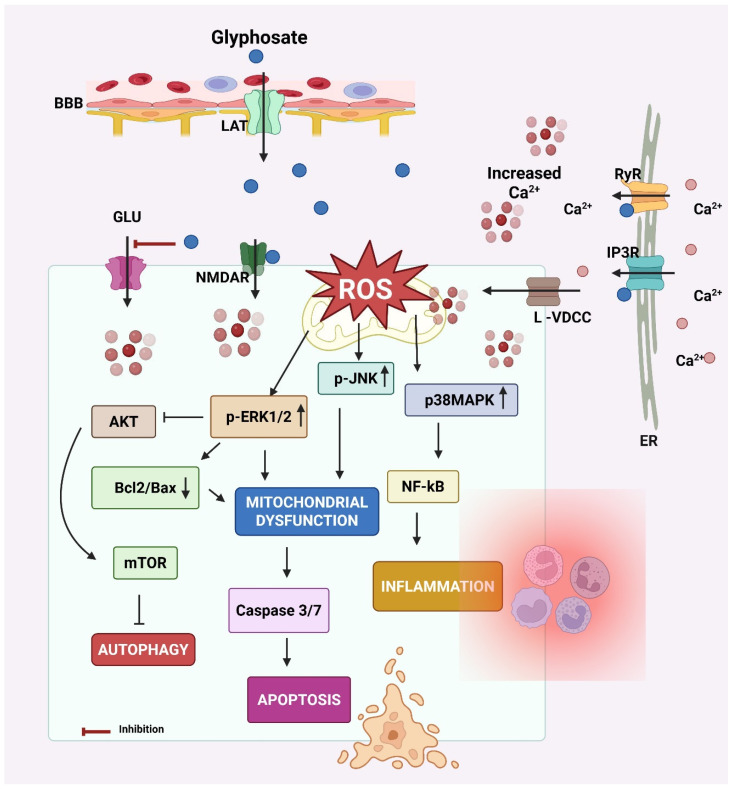
Mechanism of neurodegeneration by glyphosate. Glyphosate crosses the blood–brain barrier (BBB) via the L-type amino acid transporter (LAT). Glyphosate increases intracellular calcium (Ca^2+^) levels by activating N-methyl-D-aspartate receptors (NMDAR) and L-type-voltage-dependent calcium channels (L-VDCC) at the cell membrane and inositol 1,4,5-trisphosphate (IP3R) receptors and ryanodine (RyR) at the endoplasmic reticulum (ER) membrane. The elevated intracellular Ca^2+^ results in oxidative stress by increasing reactive oxygen species (ROS) generation. ROS accumulation creates oxidative stress, a key trigger for activating MAPK signaling cascades, including extracellular signal-regulated kinases 1 and 2 (ERK 1/2) and p38 mitogen-activated protein kinase (p38MAPK). Both kinases are known to regulate cellular responses to stress, inflammation, and survival/death signals. These effects increase calcium influx from extracellular sources. Calcium signaling can activate the ERK1/2 pathway. Calcium can also modulate the protein–protein interactions of ERK1/2, influencing its subcellular localization and the repertoire of its substrates. ERK signaling pathways can influence the balance between Bcl-2 and Bax, both of which play crucial roles in cell death. Elevated intracellular Ca^2+^ and sustained ERK/p38 signaling activate NF-κB and caspases that promote inflammation and apoptosis, respectively. p38 MAPK can directly phosphorylate calcium channels, altering their structure and function, and thus affecting their ability to allow calcium ions to pass through. Additionally, ERK1/2 can influence other calcium channel subtypes, including L-type channels, which are important for neurotransmitter release in neurons. In glial cells, this cascade also contributes to neuroinflammation and release of cytokines (e.g., IL-1β, TNF-α), further harming neurons. Glyphosate also decreases glutamate (GLU) uptake by astrocytes, resulting in GLU excitotoxicity due to increased GLU in the synaptic cleft. Created by Biorender.com.

The key events leading to neurodegeneration by glyphosate have been summarized in Figure 3.

### 3.3. Agent Orange

Agent Orange (AO), a chemical herbicide, is a mixture of equal parts of two synthetic auxins, 2,4,5-T and 2,4-D. Additionally, AO contained traces of 2,3,7,8-tetrachlorodibenzo-p-dioxin (TCDD), often known as “dioxin”, a byproduct formed during 2,4,5-T synthesis. TCDD is hazardous even in trace levels, causing immediate and long-term effects. AO’s immunosuppressive, carcinogenic, and teratogenic effects are most likely brought on by TCDD toxicity [100,101]. Human exposure occurs through ingestion, inhalation, skin, or eyes. Owing to its chemical stability, AO degrades slowly in the soil. The primary constituents, 2,4-D and 2,4,5-T, are susceptible to degradation over time, particularly in specific circumstances (e.g., moisture, temperature, and the presence of microbes). However, the harmful dioxins are far more enduring and can linger in the environment for decades [102].

AO damages the plants by oversaturating them with artificial auxins, which results in unregulated cell growth that impairs the vascular system, depriving the plants of nutrients, and eventually killing them. It was used by the U.S. military as part of its herbicidal warfare program, ‘Operation Ranch Hand’, during the Vietnam War from 1961 to 1971 to clear millions of acres of forests [103]. The “orange” name comes from the color of the containers used for its storage. The chemicals are absorbed into the body when people are exposed to AO, particularly through direct skin contact, vapor inhalation, or consumption of polluted food or water. Following absorption, the substances are transported to different tissues via the bloodstream [104]. The 2,4-D and 2,4,5-T are metabolized by the liver and are subsequently eliminated in the urine. Contrarily, dioxins can build up in the adipose tissues, impacting many different biological systems, including the nervous system [105]. Several epidemiology studies have linked these components of AO to cancer, respiratory, endocrine, and neurological diseases [106]. Epidemiological studies on AO exposure and its association with AD and PD have shown mixed results. The report published by the US Health and Medicine Division indicated suggestive but limited evidence that exposure to AO utilized during the Vietnam War correlates with a higher likelihood of developing PD [107]. AO has been known to exert toxic and degenerative effects on the human nervous system, as observed in a Korean Vietnam veterans study group. The adjusted ORs were significantly highest for AD (OR 1.64), followed by systemic atrophies affecting the nervous system, including spinal muscular atrophy (OR 1.27), and peripheral polyneuropathies (OR 1.09) [106], besides increased risk for all types of dementia [108]. Martinez et al. [109] found that veterans exposed to AO had nearly double the prevalence of dementia. While aging may be a confounding factor, the exposed group showed accelerated brain atrophy in the bilateral frontal (especially, the ventrolateral prefrontal area) and temporal lobes, compared with the age-matched control group [110], implying an effect beyond physiological aging. One retrospective study showed significant differences in the hemispheric Unified Parkinson’s Disease Rating Scale (UPDRS) between patients with and without AO exposure [111].

#### 3.3.1. BBB Permeability

It has been shown that both 2,4-D and 2,4,5-T can enter the brain by crossing the BBB and blood-CSF barrier (BCSFB) via specific organic anion transporters (OATs) [112]. The transport requires energy and is blocked by ouabain (which blocks Na^+^/K^+^ ATPase) and phlorizin (a glucose transport inhibitor) [113,114]. 2,4-D is a substrate of OAT3 in the choroid plexus. However, high concentrations of 2,4-D can inhibit this clearance mechanism, leading to its accumulation in the brain and CSF [115].

#### 3.3.2. Mechanism of Neurodegeneration in AD

Earlier research demonstrated that 2,4-D exhibited lipotoxic effects on Schwann cells, myelin, and gangliosides [116], which are essential for effective brain function and conductivity. Researchers investigated the effect of short-term exposure to 2,4-D or 2,4,5-T (constituents of AO) in human CNS-derived neuroepithelial cells (PNET2) [117]. The exposure significantly impaired mitochondrial function, induced degenerative morphological changes, and reduced cell viability. Correspondingly, glyceraldehyde-3-phosphate dehydrogenase (GAPDH) expression was significantly inhibited while 4-hydroxy-2-nonenal (HNE), a marker of lipid peroxidation, was increased. Exposure to 2,4,5-T progressively increased tau phosphorylation. A similar, though less pronounced, effect was seen with 2,4-D. Both herbicides significantly elevated amyloid precursor protein (APP) and Aβ levels. Finally, increased choline acetyltransferase (ChAT) and decreased AChE expression by the herbicides altered cholinergic function significantly [117]. In TCDD-treated human neuroblastoma cells, transcriptional (binding to xenobiotic response elements, crosstalk with other transcription factors) and post-transcriptional (microRNA, mRNA stability, alternate splicing) regulatory mechanisms participate in AhR-mediated dysregulation of AChE [118].

In a recent study, the effect of 2,4-D, 2,4,5-T, or both (D+T) was evaluated on AD biomarker expression using rat brain slices [119]. Histopathological changes included degeneration of neurons, white matter, and endothelial cells, as well as molecular/biochemical abnormalities that indicated cytotoxic injury, lipid peroxidation, DNA damage, and increased immunoreactivity to GFAP, activated Caspase 3, phosphorylated tau, Aβ, and ChAT. The combined effect (D+T) was more pronounced than the single component [119]. This study suggested that AO exposures cause molecular and metabolic alterations in frontal lobe brain tissue that resemble diseases linked to early-stage AD-type neurodegeneration. Also, AD patients exposed to AO had significantly greater levels of plasma Aβ oligomer than the control and AD patients without AO exposure, suggesting that AO might affect the AD pathology via a different mechanism [120].

Dioxin exposure disrupts granule cell neurogenesis [121] and might lead to dementia and AD through interconnected pathways. It strongly activates the aryl hydrocarbon receptor (AhR), which leads to widespread changes in gene expression [122]. This activation results in the induction of oxidative stress and neuroinflammation, demyelination [123], increased production of neurofilaments, decreased AChE [124], and several other genetic/epigenetic changes [125]. Studies have shown white matter changes in the brain after dioxin exposure as seen by reduced fractional anisotropy (FA) in the cingulum hippocampal region (CGH) and the right uncinate fasciculus (UNC) [126]. These regions are crucial for memory, cognition, and emotional processing; hence, dioxin exposure largely results in mild cognitive impairment (MCI) and AD.

Aging itself poses a significant risk for AD, marked by increased oxidative stress, dysfunctional mitochondria, weak detoxification mechanisms, and reduced neuroplasticity. These age-related vulnerabilities can act synergistically with environmental toxins such as dioxin and AO components, enhancing tau hyperphosphorylation, amyloid build-up, and synaptic dysfunction, accelerating the AD-like pathology. Thus, aging is a key modifier of chemical-induced AD phenotypes and should be considered an interacting risk factor in environmental neurotoxicity models [127].

#### 3.3.3. Mechanism of Neurodegeneration in PD

To elucidate the effects of AO exposure on PD, the clinical characteristics and 18F-N-(3-fluoropropyl)-2β-carboxymethoxy-3β-(4-iodophenyl) nortropane (FP-CIT) positron emission tomography (PET) uptake between patients with AO exposure and patients with no exposure were investigated [111] though it did not account for lifestyle factors. The AO-exposure significantly increased stiffness and tremors and decreased facial expression scores in the patients than the control. All basal ganglia areas displayed reduced 18F-FP-CIT uptake and a higher asymmetry index of anterior and posterior putamen in patients exposed to AO [111]. This finding suggests the possibility of a different pathophysiology of PD in patients exposed to AO from idiopathic PD. The PD incidence was 1.68 times greater when AO exposure and drug-induced parkinsonism (DIP)-risk drug usage were combined, and 1.31 times higher when AO exposure was used alone. The findings imply that individuals with AO exposure should be closely monitored, especially when on DIP-risk medications [128].

Previously, Bortolozzi et al. [129] studied the effect of 2,4-D in rats and observed a reduction in serotonin, dopamine, and their metabolites in the brain. Such disruptions can worsen PD pathology by contributing to both motor (bradykinesia and rigidity) and non-motor (depression and fatigue) features [130]. The 2,4-D’s neurotoxic effects can be related to the dopaminergic system via microglial activation [131]. It also influenced monoamine (MAO) activity in adult rats’ basal ganglia [132] and caused reversible changes in dopamine (D2-type) receptors [129,133]. Moreover, chronic exposure to 2,4-D affected object recognition memory in rats [134,135]. Of special interest is that 2,4-D reduces levels of white matter sulfatides that are linked to cognitive-motor dysfunction [136]. Additionally, ceramide conversion to glucosyl- or galactosylceramide is the intermediate process in sphingomyelin biosynthesis, which is inhibited by 2,4-D. Sphingomyelin has a critical role in the maintenance of the plasma membrane and myelin [136]. Higher doses and longer exposure durations disrupted brain lipid expression by lowering gangliosides, needed for inter-neuronal communication and connectivity [137]. Alterations in lipid expression, particularly of sulfatides and gangliosides, compromise myelin stability and neuronal signaling, both crucial for motor regulation. In aged rats, 2,4-D reduced locomotion and rearing and enhanced immobility in the open-field test, effects that corresponded with reduced striatal content of serotonin. These effects were reversed by treatment with 5-hydroxytryptophan (5-HTP), a precursor to serotonin, improving locomotion and rearing frequencies and diminishing immobility period [135], suggesting serotonergic restoration alleviated some motor impairments.

In the Agricultural Health Study (AHS) cohort, incident PD and the use of various pesticides, including 2,4,5-T, were studied. The results indicated that 2,4,5-T increased PD risk (Hazard ratio [HR]: 1.57, 95% confidence interval [CI]: 1.21–2.04) [138]. These findings were consistent with the earlier AHS-wide analysis based on 78 self-reported incident PD cases [139]. A case–control study reported that PD risk was tripled in a person exposed to the 2,4-D (OR: 2.59; 1.03–6.48), compared to no exposure [140].

**Figure 4:**
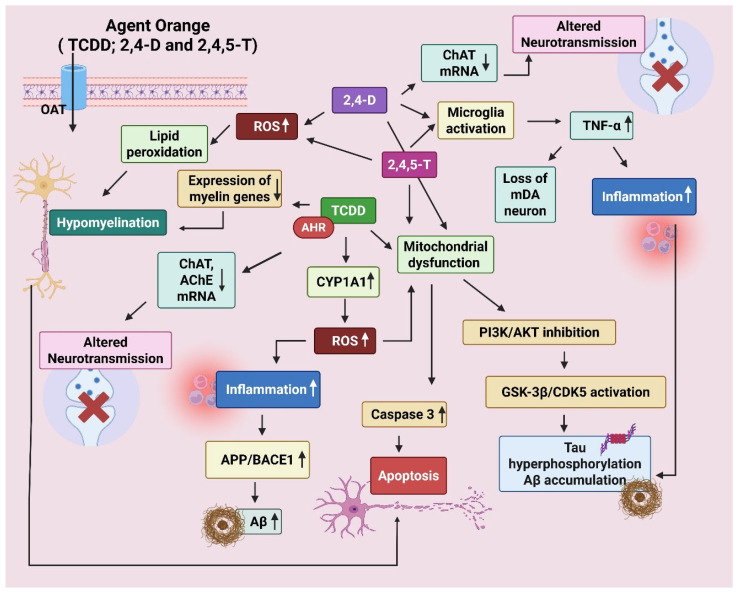
Mechanism of neurodegeneration shown by Agent Orange (AO). AO is a cocktail of 2,3,7,8-Tetrachlorodibenzo-p-dioxin (TCDD), 2,4-Dichlorophenoxyacetic acid (2,4-D), and 2,4,5-Trichlorophenoxyacetic acid (2,4,5-T). The chemicals enter the brain through organic anion transporters (OATs). TCDD disrupts oligodendrocyte development via aryl hydrocarbon receptor (AhR) activation. It decreases the expression of myelin-producing genes, resulting in hypomyelination. It also reduces choline acetyltransferase (ChAT) and Acetylcholinesterase (AChE) expression via AhR and cytokine-mediated signaling. TCDD forms DNA adducts via AhR-CYP1A1, resulting in oxidative damage (ROS). 2,4-D/2,4,5-T leads to mitochondrial dysfunction, caspase-3 activation, and apoptosis. Increase APP (amyloid precursor protein) and β-secretase (BACE1), enhance amyloid beta (Aβ) production. Tau phosphorylation is promoted by activation of GSK-3β (glycogen synthase kinase 3β) and CDK5 (cyclin-dependent kinase 5). TCDD-induced oxidative stress and cytokines can activate GSK-3β via inhibition of the PI3K/Akt pathway. Created by Biorender.com.

The mechanism of neurodegeneration by AO and its components has been illustrated below (Figure 4).

### 3.4. Ammonium Chloride

Ammonium chloride (NH_4_Cl) is commercially used as a fertilizer to improve soil structure and provide nitrogen to plants. It can also be used to make the soil more acidic, which is beneficial for plants thriving in acidic soil. When used selectively, it can serve as a weed-control tool. Excess ammonium ions cause chlorosis, leaf burn, and stunted growth, thereby killing the weed [141]. Sometimes it is mixed with glyphosate or other herbicides for a better effect. Nearly 90% of NH_4_Cl is used in the production of various compound fertilizers. Its overuse affects soil health by increasing acidity and also contributes to water pollution. It can enter the human body through inhalation, ingestion, and skin contact [142]. Once inside the body, it dissociates into ammonium (NH_4_^+^) and chloride (Cl^−^) ions. The ammonium ion is converted into urea in the liver and excreted through the kidneys in urine. When taken in large amounts, it can lead to a variety of health issues like asthma, gastritis, stomach ulcers, dermatitis, and renal toxicity [143].

#### 3.4.1. BBB Permeability

The systemic administration of NH_4_Cl leads to the formation of ammonia (NH_3_), which can readily cross the BBB. At physiological pH (~7.4), the major fraction of ammonia (99%) appears as NH_4_^+^, and the rest appears as NH_3_. Early tracer studies suggested that from plasma, over 20% of ammonia is transported as NH_4_^+^ to the brain [144], while in other locations (astrocytes) protein-mediated transport of NH_4_^+^ was noticed. To a certain extent, NH_4_^+^ can compete with K^+^ and use K^+^ transporting proteins for the movement [144]. Other findings indicated the importance of the blood pH gradient for the forward flux of ammonia from the blood into the brain [145]. It can also influence ion channels, glutamine transporters, vacuolar-type H^+^-ATPase (V-ATPase), and aquaporins (AQP-4), contributing to neurotoxicity, excitotoxicity, and cerebral edema [146,147,148]. These alterations lead to excitotoxicity by hindering astrocytic glutamate absorption and enhancing NMDA receptor overactivation, resulting in calcium overload, oxidative stress, and neuronal injury [149,150]. The main signs of this toxicity involve elevated intracellular Ca^2+^, mitochondrial impairment, and increased ROS [150]. Severe BBB dysfunction is characterized by the loss of tight junction proteins (e.g., claudin-5), enhanced permeability due to MMP-9 activation, and astrocyte swelling [151]. Astrocyte swelling leads to severe inflammation due to the release of several pro-inflammatory cytokines, including TNF-α, IL-1β, and IL-6 [152]. Thus, high levels of ammonia are neurotoxic and can cause severe BBB dysfunction, mitochondrial damage, neuroinflammation, and cognitive decline [153,154].

#### 3.4.2. Mechanism of Synaptic Dysfunction

Synaptic dysfunction is a common hallmark of both AD and PD, and NH_4_Cl has been widely used to model certain aspects of this pathology. Acute exposure to NH_4_Cl (5 mM) has been shown to reversibly decrease excitatory synaptic transmission at the CA3–CA1 synapses in the mouse organotypic hippocampal coinciding with depolarization of astrocytes and altered astrocytic passive membrane properties, but without affecting CA1 neuronal membranes. Crucially, the inhibition of astrocytic glutamine synthetase prevents this synaptic suppression, highlighting the key role of astrocyte metabolism in maintaining neurotransmission [146]. These acute effects, largely attributed to NH_4_Cl-induced intracellular alkalization, disrupt ion gradients and promote transient synaptic vesicle (SV) release [155,156]. While such changes may not directly drive Parkinsonian degeneration, prolonged dysregulation of vesicle trafficking and α-synuclein homeostasis has been implicated in dopaminergic neuron vulnerability in PD [157,158]. A study documented that NH_4_Cl changed the passive membrane properties of astrocytes, suggesting that the altered astrocytic function affects synaptic transmission [146]. In parallel, earlier studies using CA1 pyramidal neurons in acute rat hippocampal slices have demonstrated that low concentrations of NH_4_Cl (1–4 mM) depolarized the neurons by ~15 mV and severely impaired synaptic potentials, with recovery times of 10–15 min after NH_4_Cl washout [159].

As discussed above, these acute effects are largely attributed to NH_4_Cl-mediated intracellular alkalization, which disrupts ion gradients, leads to membrane depolarization, and triggers intracellular calcium overload, ultimately interfering with synaptic vesicle release and pH-sensitive signaling [155]. Elevated ammonia levels can activate astrocytes and microglia, leading to the release of inflammatory cytokines and promoting neuroinflammation, which may contribute to chronic neuropathology [160]. While these findings outline early astrocyte- and neuron-driven dysfunction, the long-term or chronic effects of NH_4_Cl remain poorly characterized.

#### 3.4.3. Mechanism of Neurodegeneration in AD

Earlier research has shown that NH_4_Cl exposure induced mitochondrial permeability transition (MPT), a mechanism related to mitochondrial dysfunction and often brought on by oxidative stress [161]. Increased ammonia levels raised mature APP in astrocytes, elevating Aβ production. This resulted from the increased endocytosis of APP into the endoplasmic reticulum, where BACE1 and γ-secretase enzymes produce Aβ_42_ [162]. Aβ aggregates interact with glutamatergic neurotransmission, which weakens excitatory synaptic plasticity, resulting in cognitive decline [163]. Significant microglial activation, neuroinflammation, and cell loss were observed in the astrocyte-microglia co-culture model after NH_4_Cl incubation [164]. Autophagy and lysosomal pathology are inextricably linked in AD as the autophagy-lysosomal pathway (ALP) helps in eliminating misfolded proteins and other cellular waste. NH_4_Cl is a lysosome inhibitor that reduces autophagic flux by increasing the level of misfolded proteins, mitochondrial stress, and neuronal loss [165]. Disruption of autophagic flux leads to toxic protein accumulation and neuronal damage, which accelerates AD progression.

#### 3.4.4. Mechanism of Neurodegeneration in PD

NH_4_Cl inhibited lysosome-mediated degradation, resulting in the cytosolic buildup of α-synuclein and myocyte enhancer factor 2D (MEF2D) proteins in a human dopaminergic neuroblastoma SH-SY5Y cell line. It lowered MEF2D expression in the nucleus, consequently diminishing its protective role against mitochondrial dysfunction and oxidative stress [166]. In an earlier study, NH_4_Cl (16 mM) excessively stimulated acid-sensitive ion channels (ASICs) in dopamine neurons of the midbrain. The depolarization and continuous firing of action potentials may lead to neuronal dysfunction or injury, as in PD [167]. Cultured astrocytes exposed to ammonium exhibited impaired function of the 5-hydroxytryptamine receptor 2B (5-HT_2_B) as evidenced by a loss of 5-HT-induced Ca^2+^ signaling and ERK1/2 phosphorylation. Elevated ammonium also increases the activity of the RNA-editing enzyme, adenosine deaminase acting on RNA 2 (ADAR2), resulting in aberrant RNA editing of 5-HT_2_B mRNA contributing to astrocytic dysfunction [168]. Given the interaction of 5-HT_2_B with dopaminergic neurons, such dysregulation might influence motor control, as seen in PD.

**Figure 5:**
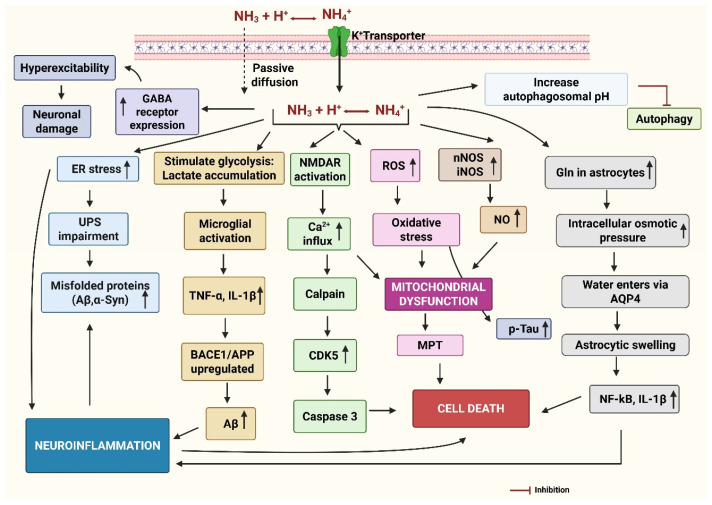
The mechanism of neurodegeneration by ammonia. Ammonia is present as NH_3_ at physiological pH, which readily diffuses in the brain and extracellular space. NH_4_^+^ is transported via potassium (K^+^) transporters. The oxidative stress activates the microglia, resulting in the release of pro-inflammatory cytokines, increased amyloid precursor protein (APP) processing and beta-secretase (BACE1) activity to generate amyloid-beta (Aβ), increased phosphorylation of tau, and aggregation of alpha-synuclein (α-Syn). Elevated ammonia generates oxidative stress by producing reactive oxygen species (ROS) and generating nitric oxide (NO) by neuronal nitric oxide synthase (nNOS) and inducible nitric oxide synthase (iNOS). ROS and excessive NO are the key sources of mitochondrial dysfunction, induction of mitochondrial permeability transition (MPT), and impaired energy metabolism both in neurons and astrocytes. Activation of NMDA receptors leads to the opening of the ion channel, allowing increased Ca^2+^ ions into the post-synaptic neuron, causing mitochondrial ROS, and activating calpain, which can induce neuronal death directly. Activated calpain also activates cyclin-dependent kinase (CDK5) and caspase3, causing neuronal death. Ammonia can increase the rate of cerebral glucose utilization and oxygen consumption, leading to increased lactate release from the brain, particularly in the astrocytes. Elevated lactate can activate microglia and release inflammatory cytokines like tumor necrosis factor-alpha (TNF-α) and Interleukin-1 beta (IL-1β). ER stress can impair the unfolded protein response (UPR), causing protein misfolding and accumulation, contributing to inflammation. Excess ammonia increases levels of glutamine (Gln) in the astrocytes, which eventually leads to astrocytic swelling due to influx of water through aquaporin (AQP4) channels and osmotic lysis. Ammonia can increase the expression of gamma-aminobutyric acid (GABA) receptors, further contributing to enhanced GABAergic transmission. This can contribute to neurodegeneration by increasing inhibitory neurotransmission, disrupting energy metabolism, and promoting inflammation. Ammonia also increases the autophagosome pH and inhibits autophagy. Created by Biorender.com.

The main events in the neurodegeneration caused by NH_4_Cl have been presented in Figure 5.

While ammonium chloride triggers molecular and neuronal changes resembling AD and PD in lab studies, there is no epidemiological evidence in human populations to support it as a risk factor. Therefore, more studies on human populations are needed in this direction.

Below is a comparative table (Table 1) summarizing key characteristics of common gardening chemicals concerning their chemical nature, molecular weight, routes of human exposure, transport across the BBB, mitochondrial entry mechanisms, and their modes of action in AD and PD pathogenesis.

## 4. Effect of Common Gardening Chemicals on Gut–Brain-Axis

The gut–brain axis represents a complex network of communication systems between the gut and the brain, comprising the enteric nervous system, autonomic nervous system, and the immune system. Disruptions in the gut–brain axis can impact brain health and contribute to neurodegenerative processes. Gut dysbiosis can disrupt the production of neurotransmitters like serotonin, which plays a role in mood and cognitive function. In addition, gut dysbiosis can trigger inflammation in the gut, which can then spread to the brain via this axis, contributing to neuroinflammation [171].

Exposure to PQ and glyphosate has been linked to disruptions in the gut microbiota, which may contribute to the development of neurodegenerative diseases such as AD and PD. Glyphosate exerts an inhibitory effect on microbial EPSP synthase, mainly affecting beneficial bacteria. On the contrary, Clostridium and Salmonella are resistant to it, suggesting the role of glyphosate in causing dysbiosis [148]. The overgrowth of bacteria such as Clostridia generates high levels of toxic metabolites in the brain, contributing to the development of neurological deviations. Glyphosate’s disruption of manganese homeostasis selectively affects Lactobacillus and can lead to several disorders, such as PD [149]. Although the mechanism remains uncertain, it could be correlated to dysbiosis. Intestinal dysbiosis in PD patients causes an immunological stimulation and may contribute to α-synuclein misfolding [150]. If dysbiosis results in endotoxemia (leaky gut), infiltrated bacterial by-products such as lipopolysaccharides (LPS) could initiate inflammatory pathways [99]. Human gut dysbiosis is also linked to AD etiology in animals exposed to Gly, where there is an imbalance in the intestinal bacterial composition. AD patients showed a reduced level of both Firmicutes and Bifidobacteria and an increase in Bacteroidetes [172,173]. Pseudomonas break down glyphosate to phosphate and carbon (for amino acid synthesis) and formaldehyde (toxic by-product) [174], and low levels of formaldehyde can induce amyloid-like misfolding of tau protein in neurons, forming protein aggregates similar to those observed in association with AD [175]. Moreover, a correlation was noted between an abundance of Escherichia and Shigella in the guts of AD individuals and brain amyloidosis and behavioral impairment [152]. Additionally, elevated levels of LPS have been found in the brain and plasma of patients with AD [153]. Lower levels of sulfate in the brain have been associated with neurological diseases, especially AD and PD [176]. Glyphosate might block the movement of sulfate from the gut to the liver and also interfere with the body’s ability to make sulfate in blood vessels and blood cells. Over time, this could cause a serious lack of sulfate in many parts of the body [177,178]. Similarly, PQ exposure in a mouse model resulted in gut dysfunction by disrupting the gut microbiota, damaging the intestinal epithelial barrier, and activating the inflammatory cascade. Additionally, the presence of α-synuclein aggregates in the colon and midbrain, and the altered metabolism of short-chain fatty acids (SCFAs), was reported [179]. This suggests that pathological α-syn and SCFAs from the gut may be significant components of the gut–brain axis. Exposure to 2,4-D and dioxin-like compounds shifted the Firmicutes-to-Bacteroidetes ratio to a dysbiosis one and affected the metabolism of urea, amino acids, and carbohydrates [180,181] and caused a systematic deficiency of essential vitamins [182]. However, there are not any research studies evaluating their impact on gut microbiota and resultant effects on the gut–brain axis in the context of AD and PD. While direct evidence linking ammonium chloride to AD or PD via the gut–brain axis is limited, the neurotoxic effects of its metabolite, ammonia, suggest a potential indirect role [183]. Further research is needed to fully understand these connections and their implications for neurodegenerative diseases.

## 5. Regulatory Overview of the Selected Gardening Chemicals

According to the present legal status and international regulatory frameworks concerning the compounds, PQ has been banned in over 70 countries due to health risks, yet it remains in use in the United States (US) [184]. A critical review based on the studies published between 2006 and 2021 found no conclusive evidence between PQ exposure and PD [69]. In its 2021 interim decision, the Environmental Protection Agency (EPA) announced “insufficient evidence to link PQ exposure to PD”, and as of February 2025, the US EPA has not banned the herbicide PQ [185]. Nonetheless, recent studies have suggested an association between PQ exposure and an increased risk of PD, promoting renewed scrutiny and calls for regulatory action. The forthcoming EPA report is expected to offer a revised assessment of the potential health risks linked to PQ [186].

Based on early toxicological evaluations, the US EPA classified glyphosate in the lowest toxicity category (category IV), considering it essentially non-toxic and non-irritating [187]. However, growing evidence indicates that glyphosate exhibits cytotoxic and genotoxic properties, promotes oxidative stress, and may be carcinogenic. In light of these concerns, the International Agency for Research on Cancer (IARC) classified glyphosate as “probably carcinogenic to humans” [188]. In 2020, the EPA recommended updating product labels to reduce harm, including clearer spray drift warnings and consistent labeling. However, after a court challenge in 2022, the EPA withdrew these recommendations [189]. Meanwhile, Bayer announced it would stop selling glyphosate-based herbicides for home use in the U.S. by 2023 due to mounting lawsuits [190]. Despite this, glyphosate products remain on the market with no label changes. In 2024, the EPA rejected a petition to ban glyphosate, stating there was no new evidence of increased toxicity [191]. The European Chemicals Agency (ECHA) determined that glyphosate is not a carcinogen, yet it can result in significant eye injury and is harmful to aquatic organisms. Also, the European Food Safety Authority (EFSA) found “no critical areas of concern” for human, animal, and environmental health from the use of glyphosate in agriculture [192]. As a result, in 2023, the European Commission (EC) approved the renewal of glyphosate’s authorization for an additional ten years [193].

While the original AO mixture has been banned, its components, especially 2,4-D, are still used. Global health and safety agencies, including the World Health Organization (WHO), EC, U.S. EPA, Health Canada, and New Zealand authorities, have reviewed 2,4-D and have found it noncarcinogenic in animal studies [194]. Regulatory oversight of 2,4-D varies globally, as it is banned in some countries due to potential health and environmental effects [194], but the U.S. EPA allows its continued use with certain restrictions [195]. Lastly, the use of ammonium chloride is regulated under general chemical safety and environmental protection laws [196]. It is not presently categorized as a neurotoxic chemical, but it is regulated by occupational exposure limits and assessments of environmental hazards [197].

## 6. Conclusions

In conclusion, there is growing evidence linking common gardening chemicals to the development of neurodegenerative disorders like AD and PD. Despite being useful for removing weeds and encouraging plant growth, chemicals such as PQ, glyphosate, AO, and NH_4_Cl can be extremely neurotoxic if overused or improperly handled. These chemicals can remain in soil, water, and plant matter, resulting in pollution across various trophic levels. When they enter the food chain, they can cause the buildup of toxic substances in higher organisms, impacting the health of wildlife as well as human populations that eat contaminated crops or livestock products. For instance, PQ and NH_4_Cl infiltrate groundwater, affecting surrounding aquatic ecosystems. Traces of glyphosate were reported in several foods (cereals, honey, meat, and milk) in quantities that could lead to long-term health hazards, especially with prolonged exposure. Likewise, dioxins are very stable and often accumulate within the food chain, mainly in fatty tissues. These chemicals are associated with a higher risk of neurodegenerative diseases, despite some controversial findings. The mechanism of action involves glutamate excitotoxicity, dopaminergic pathway disruption, oxidative stress, mitochondrial dysfunction, neuroinflammation, and synaptic dysfunction, crucial in the pathophysiology of AD and PD. Even with increasing evidence connecting these herbicides to neurodegenerative disorders, regulatory bodies like the U.S. EPA have not consistently prohibited them. Ongoing discussion surrounds their safety, underscoring the necessity for more definitive epidemiological and mechanistic research. These incidents highlight the need for stricter laws and environmentally friendly agricultural practices to lessen the effects of these toxins on the environment and food supply. In addition, regulatory and policy recommendations should be strengthened and include (i) reclassification of neurotoxic herbicides under stricter hazard categories, (ii) mandatory neurotoxicity testing as part of pesticide registration, (iii) labeling requirements highlighting risks to neurological health, and (iv) phase-out plans for high-risk substances, especially in residential or gardening contexts. Also, environmental/human monitoring should be expanded to track chronic low-dose exposure and its links to neurodegeneration. Long-term research and an improved comprehension of exposure trends are crucial for guiding public health policies and minimizing the risk of neurotoxic environmental exposure. Additional research is essential to clarify the specific mechanisms of action and create preventive measures to reduce these risks.

While numerous studies highlight the acute neurotoxic effects of gardening chemicals, far less is known about their long-term impact, especially from chronic low-dose exposure. Moreover, long-term exposure may not only increase the risk of neurodegeneration but also affect reproductive health, hormone regulation, and immune function. Environmentally persistent residues of these chemicals can alter soil microbiota and reduce biodiversity, with downstream effects on human health. Notably, there are no reported epidemiological studies evaluating the association between NH_4_Cl exposure and increased risk of neurodegenerative diseases, despite substantial experimental evidence. Lifestyle factors (such as physical activity, diet, smoking, and occupational exposures) and aging can significantly influence the development and progression of AD and PD. Therefore, future studies (longitudinal, case–control, and biomarker-based) that include such cofounders are strongly recommended to investigate the potential neurotoxic effects of this chemical in human populations, especially in vulnerable populations such as farmers, gardeners, and children living near treated areas.

## Figures and Tables

**Table 1:** Mode of action of some common gardening chemicals in AD and PD.

Chemical	Disease	Study	Dose/Treatment	Effect	Mechanism	Refs.
Paraquat	AD	APP/PS1 mice	10 mg/kg; i.p./3 weeks	Cognition impairment, elevated Aβ levels, NLRP3 inflammasome activation	Oxidative stress, Inflammation	[48]
		APP transgenic mice	10 mg/kg; i.p./4 weeks	Impaired associative learning and memory, and increased Aβ levels	Oxidative stress	[47]
		C57BL/6J mice; isolated NPCs	40 to 120 μM	Wnt/β-catenin signaling pathway inhibition; Reduced cellular β-catenin, p-GSK-3β, and cyclin-D1 and increased Bax/Bcl2	Oxidative stress	[49]
		C57BL/6J Mice	1.25 mg/kg; i.p./3 weeks	Negatively affected adult hippocampal neurogenesis and cognitive function; Reduced AKT phosphorylation	Oxidative stress, Inflammation	[52]
		hNPCs	100 μM	Downregulated Wnt signaling pathway via miRNA in hNPCs	Oxidative stress, decreased activity of glutamate receptors	[50]
	PD	C57BL/6 Mice	10 mg/mL/oral/4 months	Decreased SOD, GSH-PX; Increased MDA and GSSG; Decreased number of TH positive neurons and expression of DAT	Oxidative stress	[54,169]
		Human neuroblastoma SK-N-MC cell line	100–500 μM	Trx1 oxidation; Activation of JNK and caspase 3; Death of the dopaminergic neurons	Oxidative stress, Apoptosis	[170]
		C57BL/6 Mice	10 mg/kg/i.p./once a week/3 consecutive weeks	Increased α-synuclein production and aggregation	Oxidative stress, Inflammation	[58]
		C57BL/6 Mice; Rat dopaminergic neural cell line N27	10 mg/kg/i.p./once/every 3 days	Increased Nox1	Oxidative stress	[60]
		Wistar rats	10 mg/kg/2 mL/i.p./once a week/37 weeks	Altered levels of GSK-3β in the midbrain and striatum	Defective signaling pathway	[64]
	AD/PD	Sprague Dawley rats; PHBME cells	30 mg/kg/i.p/twice per 3 weeks; 80 μM	Over-activated PI3K/AKT signaling; Increased IL-6 production; BBB dysfunction	Oxidative stress, Inflammation	[55]
		C57BL/6J mice; MSC80 cell line	10–30 mg/kg/i.p./1 week; 100 μM	Demyelination	Oxidative stress, Inflammation	[44,46]
Glyphosate	AD	C57BL/6J mice; APP/PS1 primary cortical neurons	125–500 mg/kg/oral gavage/14 days	Elevated TNFα and soluble Aβ_40–42_; Cytotoxicity; Disrupted transcriptome	Inflammation	[77]
		3xTg-AD mice	50 or 500 mg/kg daily/13 weeks, followed by a 6-month recovery period	Elevated Aβ and tau pathology and worsening spatial cognition after recovery; Increased inflammatory cytokines	Inflammation	[83]
		Wistar rats	0.38% orally during pregnancy and lactation (till 15-day-old); Hippocampal slices	NMDA receptor overstimulation; Mitochondrial dysfunction, Ca^2+^-mediated neuronal damage; Activation of ERK and CaMKII	Glutamatergic excitotoxicity and oxidative damage	[81]
		Wistar rats	400 mg/kg/oral/single dose	Increased excitatory amino acids	Excitotoxicity	[82]
	PD	C57BL/6 mice	10 mg/kg/14 days	Dopaminergic neurotoxicity in the striatum and SNr	Oxidative damage	[87]
		Wistar rats	0.36% at gestational day 5 (GD5) and continually up to postnatal day 15 (PND15) or up to postnatal day 60 (PND60)	NMDA receptor activation, impairment of cholinergic transmission; Astrocyte dysfunction; ERK1/2 overactivation; Decreased NF-κB phosphorylation	Glutamatergic excitotoxicity and oxidative damage	[80]
		Neuronal-differentiated PC12 cells	10–40 mM	Activated autophagy and apoptotic pathways	Apoptotic and autophagic cell death	[90]
	AD/PD	Sprague Dawley albino rats; Hippocampal slices	16.9 mg/kg/i.p.; 1 μM and 100 μM	Inhibited LTP and learning	Inflammation	[96]
Agent Orange/Components	AD	Human CNS-derived neuroepithelial cells	250 μg/mL (2,4-D and 2,4,5-T)	Increased Aβ, tau phosphorylation, and lipid peroxidation; Increased ChAT; Decreased AChE	Oxidative damage, Cytotoxicity	[117]
		Long-Evans rat frontal lobe slice cultures	250 μg/mL (2,4-D;2,4,5-T, or both)	Cytotoxic injury; Lipid peroxidation, DNA damage; Increased immunoreactivity to activated Caspase 3, GFAP, phosphorylated tau, Aβ, and ChAT	Oxidative damage, Cytotoxicity	[119]
		Wistar Rat	9.28 × 10^−3^ g per hectare (g.a.i/ha)/oral and nebulize (2,4-D)	Impairment of exploration and recognition memory	Oxidative stress	[134]
		Human neuroblastoma SK-N-SH cell line	0.1–10 nM (Dioxin)	Suppressed neuronal AChE via AhR-mediated transcriptional down-regulation	Cholinergic neurotransmission	[124]
		Plasma		Higher plasma Aβ oligomer	Amyloidosis	[120]
	PD	Patients with Agent Orange exposure	Clinical profile and FP-CIT PET findings	Lower facial expressions, tremors, and rigidity; Significantly lower caudate/putamen ratios		[111]
		C57BL/6 mice	30 μg TCDD/Kg/i.p.	AhR-mediated induction of CYP1A1; Activation of NF-kB; Selective dopaminergic neuronal damage	Oxidative stress, Inflammation	[121]
		Wistar rats	70 mg/kg/day of 2,4-D from gestation day (GD) 16 to postpartum day 23	Adverse neuronal changes (in D2 receptors, tyrosine hydroxylase, and dopamine beta-hydroxylase) and behavioral changes	Decreased DA neurotransmission	[129,133]
		Wistar rats	100 and 200 mg/kg/oral of 2,4-D	Behavioral changes and neurochemical parameters	Dopamine dysfunction	[135]
		Retrospective cohort study		1.31 times higher PD risk		[128]
		AHS		2,4,5-T positively associated with increased PD risk (HR: 1.57, 95%CI: 1.21–2.04)		[138]
Ammonium Chloride	AD	Cortical astrocytes from a rat	10 mM	Elevated APP expression; Increased Aβ production	Amyloidogenesis	[162]
		Cultured astrocytes	5 mM	Mitochondrial permeability transition	Oxidative stress	[161]
		Primary rat glial co-cultures of astrocytes	20 mM	Microglial activation; Cell death	Inflammation	[164]
		Rat hippocampal cultures	5–50 mM	Increased [Ca^2+^]_i_	Membrane depolarization	[155]
	PD	Human dopaminergic neuroblastoma SH-SY5Y cell line	10 mM	Increased accumulation of α-synuclein and MEF2D protein	Dysfunctional lysosome-mediated degradation	[166]
		Mouse midbrain dopamine neurons	16 mM	ASICs’ gating and desensitization	Neuronal dysfunction	[167]
		Male CD-1 mice; Cultured mouse astrocytes	33 units/kg/i.p./3 days; 3 mM ammonia for 3 days	Increased ADAR2; Loss of 5-HT induced Ca^2+^ signaling and ERK1/2 phosphorylation		[168]
	AD/PD	Entorhino-hippocampal tissue cultures of mice	5 mM	Decreased spontaneous excitatory synaptic activity	Synaptic transmission dysregulation	[146]

Abbreviations. Aβ: Amyloid-beta; AChE: Acetylcholinesterase; AD: Alzheimer’s disease; ADAR2: Adenosine Deaminase Acting on RNA 2; AHC: Agricultural Health Study; AhR: Aryl hydrocarbon receptor; AKT: Protein kinase B; APP: Amyloid precursor protein; ASIC: Acid-sensitive ion channels; Bax/Bcl2: BCL2-associated X/B-cell lymphoma 2; Ca^2+^: Calcium ion; CaMKII: Ca^2+^/calmodulin-dependent protein kinase II; ChAT: Choline acetyltransferase; CI: Confidence interval; 2,4-D: 2,4-Dichlorophenoxyacetic acid; DAT: Dopamine transporter; ERK1/2: Extracellular signal-regulated kinases; FP-CIT PET: FN-(3-fluoropropyl)-2β-carbon ethoxy-3β-(4-iodophenyl) nortropane positron emission tomography; FAP: Glial fibrillary acidic protein; GSH-PX: Glutathione peroxidase; GSSG; Oxidized glutathione; GSK-3β: Glycogen synthase kinase-3 beta; hNPCs: Human Neural Progenitor Cells; HR: Hazard ratio; 5-HT: 5-Hydroxytryptamine receptor 2B; [Ca^2+^]_i_: Intracellular calcium concentration; i.p.: Intraperitoneal; JNK: c-Jun N-terminal kinases; MDA: Malondialdehyde; MEF2D: Myocyte enhancer factor 2D; MSC80: Mouse Schwann cell line; NF-κB: Nuclear factor kappa light-chain enhancer of activated B cells; NMDA: N-methyl-D-aspartate; NLRP3: NOD-like receptor family pyrin domain containing 3; NOX1: NADPH oxidase 1; NPCs: Neuronal proginator cells; PD: Parkinson’s Disease; PHBME: Primary human brain microvascular endothelial cells; SNr: Substantia nigra; SOD: Superoxide dismutase; 2,4,5-T: 2,4,5-Trichlorophenoxyacetic acid; TH:Tyrosine hydroxylase; TNF-α: Tumor necrosis factor-α; Trx1:Thioredoxin; Wnt: Wingless-type Family Member.

## Data Availability

The original contributions presented in this study are included in the article.

## References

[1] Gustavsson A., Norton N., Fast T., Frölich L., Georges J., Holzapfel D., Kirabali T., Krolak-Salmon P., Rossini P.M., Ferretti M.T. (2023). Global estimates on the number of persons across the Alzheimer’s disease continuum. Alzheimers Dement..

[2] Giménez L.B. (2024). Improving Quality of Life and Autonomy for Parkinson’s Patients: French Neurologists Selected as Finalists for the European Inventor Award 2024.

[3] Watts M. (2010). Paraquat.

[4] Maggi F., Tang F.H., la Cecilia D., McBratney A. (2019). PEST-CHEMGRIDS, global gridded maps of the top 20 crop-specific pesticide application rates from 2015 to 2025. Sci. Data.

[5] Maggi F., la Cecilia D., Tang F.H.M., McBratney A. (2020). The global environmental hazard of glyphosate use. Sci. Total Environ..

[6] Scientific T. (2022). Safety Data Shest-Ammonium Chloride.

[7] Bajgar J., Kassa J., Fusek J., Kuca K., Jun D. (2015). Other toxic chemicals as potential chemical warfare agents. Handbook of Toxicology of Chemical Warfare Agents.

[8] Blassingame H. (2021). The Past And Present of Agent Orange. https://www.interlochenpublicradio.org/2021-06-28/the-past-and-present-of-agent-orange.

[9] Mnif W., Hassine A.I.H., Bouaziz A., Bartegi A., Thomas O., Roig B. (2011). Effect of Endocrine Disruptor Pesticides: A Review. Int. J. Environ. Res. Public. Health.

[10] Corasaniti M.T., Defilippo R., Rodinò P., Nappi G., Nisticò G. (1991). Evidence that paraquat is able to cross the blood-brain barrier to a different extent in rats of various age. Funct. Neurol..

[11] Fan R., Zhang W., Jia L., Li L., Zhao J., Zhao Z., Peng S., Chen Y., Yuan X. (2021). Combined Developmental Toxicity of the Pesticides Difenoconazole and Dimethomorph on Embryonic Zebrafish. Toxins.

[12] Sasikala S., Minu Jenifer M., Velavan K., Sakthivel M., Sivasamy R., Fenwick Antony E.R. (2023). Predicting the relationship between pesticide genotoxicity and breast cancer risk in South Indian women in in vitro and in vivo experiments. Sci. Rep..

[13] Melanda V.S., Galiciolli M.E.A., Lima L.S., Figueiredo B.C., Oliveira C.S. (2022). Impact of Pesticides on Cancer and Congenital Malformation: A Systematic Review. Toxics.

[14] Panis C., Lemos B. (2024). Pesticide exposure and increased breast cancer risk in women population studies. Sci. Total Environ..

[15] Sabarwal A., Kumar K., Singh R.P. (2018). Hazardous effects of chemical pesticides on human health–Cancer and other associated disorders. Environ. Toxicol. Pharmacol..

[16] Fagundes T.R., Kawassaki A.C.B., Concato V.M., Assolini J.P., Silva T.F., Gonçalves M.D., da Silva Siqueira E., Sahd C.S., Inoue F.S.R., da Silva T.P., Rezaei N. (2022). Impact of Pesticides on Immune-Endocrine Disorders and Its Relationship to Cancer Development. Handbook of Cancer and Immunology.

[17] Masenga S.K., Kabwe L.S., Chakulya M., Kirabo A. (2023). Mechanisms of Oxidative Stress in Metabolic Syndrome. Int. J. Mol. Sci..

[18] Abdelazim A.M., Abomughaid M.M. (2024). Oxidative stress: An overview of past research and future insights. All Life.

[19] Braak H., Tredici K.D., Rüb U., de Vos R.A.I., Jansen Steur E.N.H., Braak E. (2003). Staging of brain pathology related to sporadic Parkinson’s disease. Neurobiol. Aging.

[20] Hawkes C.H., Del Tredici K., Braak H. (2007). Parkinson’s disease: A dual-hit hypothesis. Neuropathol. Appl. Neurobiol..

[21] Attems J., Walker L., Jellinger K.A. (2014). Olfactory bulb involvement in neurodegenerative diseases. Acta Neuropathol..

[22] Borghammer P., Van Den Berge N., van Laar T. (2019). Brain-First versus Gut-First Parkinson’s Disease: A Hypothesis. J. Park. Dis..

[23] Beach T.G., White C.L., Hladik C.L., Sabbagh M.N., Connor D.J., Shill H.A., Sue L.I., Sasse J., Bachalakuri J., Henry-Watson J. (2009). Olfactory bulb α-synucleinopathy has high specificity and sensitivity for Lewy body disorders. Acta Neuropathol..

[24] Matsuzaki R., Gunnigle E., Geissen V., Clarke G., Nagpal J., Cryan J.F. (2023). Pesticide exposure and the microbiota-gut-brain axis. ISME J..

[25] Kulcsarova K., Bang C., Berg D., Schaeffer E. (2023). Pesticides and the Microbiome-Gut-Brain Axis: Convergent Pathways in the Pathogenesis of Parkinson’s Disease. J. Park. Dis..

[26] Wasiak J., Zielonka K., Dądela B., Markowski M., Śliwa N., Majewska E.M., Kawalska E., Gnitecki S., Janczura S., Borowski M. (2025). Gut Microbiota and Gut-Brain Axis in Health and Disease A Narrative Review. Qual. Sport.

[27] Chen L., Yan H., Di S., Guo C., Zhang H., Zhang S., Gold A., Wang Y., Hu M., Wu D. (2025). Mapping pesticide-induced metabolic alterations in human gut bacteria. Nat. Commun..

[28] Richardson J.R., Quan Y., Sherer T.B., Greenamyre J.T., Miller G.W. (2005). Paraquat Neurotoxicity is Distinct from that of MPTP and Rotenone. Toxicol. Sci..

[29] Di Monte D., Sandy M.S., Ekström G., Smith M.T. (1986). Comparative studies on the mechanisms of paraquat and 1-methyl-4-phenylpyridine (MPP+) cytotoxicity. Biochem. Biophys. Res. Commun..

[30] Hongoeb J., Tantimongcolwat T., Ayimbila F., Ruankham W., Phopin K. (2025). Herbicide-related health risks: Key mechanisms and a guide to mitigation strategies. J. Occup. Med. Toxicol..

[31] Sørensen A.T., Ledri M., Melis M., Nikitidou Ledri L., Andersson M., Kokaia M. (2017). Altered Chloride Homeostasis Decreases the Action Potential Threshold and Increases Hyperexcitability in Hippocampal Neurons. eNeuro.

[32] Fujii T., Yokoyama E.-i., Inoue K., Sakurai H. (1990). The sites of electron donation of photosystem I to methyl viologen. Biochim. Biophys. Acta (BBA)-Bioenerg..

[33] Wang J.-W., Yang X., Ning B.-Y., Yang Z.-Y., Luo L.-H., Xiao H., Ning Z. (2019). The successful treatment of systemic toxic induced paraquat poisoning by skin absorption: Case reports and a literature review. Int. J. Clin. Exp. Pathol..

[34] Shi L., Yu G., Li Y., Zhao L., Wen Z., Tao Y., Wang W., Jian X. (2022). The toxicokinetics of acute paraquat poisoning in specific patients: A case series. J. Int. Med. Res..

[35] Sukumar C.A., Shanbhag V., Shastry A.B. (2019). Paraquat: The Poison Potion. Indian J. Crit. Care Med..

[36] Gawarammana I.B., Buckley N.A. (2011). Medical management of paraquat ingestion. Br. J. Clin. Pharmacol..

[37] Bartlett R.M., Holden J.E., Nickles R.J., Murali D., Barbee D.L., Barnhart T.E., Christian B.T., DeJesus O.T. (2009). Paraquat is excluded by the blood brain barrier in rhesus macaque: An in vivo pet study. Brain Res..

[38] Zhou Y.T., Xu Y.N., Ren X.Y., Zhang X.F. (2023). Inactivation of microglia dampens blood-brain barrier permeability and loss of dopaminergic neurons in paraquat-lesioned mice. Food Chem. Toxicol..

[39] Zhang X.-f., Thompson M., Xu Y.-h. (2016). Multifactorial theory applied to the neurotoxicity of paraquat and paraquat-induced mechanisms of developing Parkinson’s disease. Lab. Investig..

[40] Han S., Feng Y., Guo M., Hao Y., Sun J., Zhao Y., Dong Q., Zhao Y., Cui M. (2022). Role of OCT3 and DRP1 in the Transport of Paraquat in Astrocytes: A Mouse Study. Environ. Health Perspect..

[41] Rappold P.M., Cui M., Chesser A.S., Tibbett J., Grima J.C., Duan L., Sen N., Javitch J.A., Tieu K. (2011). Paraquat neurotoxicity is mediated by the dopamine transporter and organic cation transporter-3. Proc. Natl. Acad. Sci. USA.

[42] Papuć E., Rejdak K. (2020). The role of myelin damage in Alzheimer’s disease pathology. Arch. Med. Sci..

[43] Xie S., Yang J., Huang S., Fan Y., Xu T., He J., Guo J., Ji X., Wang Z., Li P. (2022). Disrupted myelination network in the cingulate cortex of Parkinson’s disease. IET Syst. Biol..

[44] Silva R., Sobral A.F., Dinis-Oliveira R.J., Barbosa D.J. (2024). The Link Between Paraquat and Demyelination: A Review of Current Evidence. Antioxidants.

[45] Bajo-Grañeras R., Sanchez D., Gutierrez G., González C., Do Carmo S., Rassart E., Ganfornina M.D. (2011). Apolipoprotein D alters the early transcriptional response to oxidative stress in the adult cerebellum. J. Neurochem..

[46] Hichor M., Sampathkumar N.K., Montanaro J., Borderie D., Petit P.X., Gorgievski V., Tzavara E.T., Eid A.A., Charbonnier F., Grenier J. (2017). Paraquat Induces Peripheral Myelin Disruption and Locomotor Defects: Crosstalk with LXR and Wnt Pathways. Antioxid. Redox Signal.

[47] Chen L., Yoo S.E., Na R., Liu Y., Ran Q. (2012). Cognitive impairment and increased Aβ levels induced by paraquat exposure are attenuated by enhanced removal of mitochondrial H_2_O_2_. Neurobiol. Aging.

[48] Chen L., Na R., Boldt E., Ran Q. (2015). NLRP3 inflammasome activation by mitochondrial reactive oxygen species plays a key role in long-term cognitive impairment induced by paraquat exposure. Neurobiol. Aging.

[49] Zhao L., Yan M., Wang X., Xiong G., Wu C., Wang Z., Zhou Z., Chang X. (2018). Modification of Wnt signaling pathway on paraquat-induced inhibition of neural progenitor cell proliferation. Food Chem. Toxicol..

[50] Yan M., Dou T., Lv W., Wang X., Zhao L., Chang X., Zhou Z. (2017). Integrated analysis of paraquat-induced microRNAs-mRNAs changes in human neural progenitor cells. Toxicol. Vitr..

[51] Jia L., Piña-Crespo J., Li Y. (2019). Restoring Wnt/β-catenin signaling is a promising therapeutic strategy for Alzheimer’s disease. Mol. Brain.

[52] Li K., Cheng X., Jiang J., Wang J., Xie J., Hu X., Huang Y., Song L., Liu M., Cai L. (2017). The toxic influence of paraquat on hippocampal neurogenesis in adult mice. Food Chem. Toxicol..

[53] Griffin R.J., Moloney A., Kelliher M., Johnston J.A., Ravid R., Dockery P., O’Connor R., O’Neill C. (2005). Activation of Akt/PKB, increased phosphorylation of Akt substrates and loss and altered distribution of Akt and PTEN are features of Alzheimer’s disease pathology. J. Neurochem..

[54] Ren J.P., Zhao Y.W., Sun X.J. (2009). Toxic influence of chronic oral administration of paraquat on nigrostriatal dopaminergic neurons in C57BL/6 mice. Chin. Med. J..

[55] Liu T., Zheng F., Liu L., Zhou H., Shen T., Li Y., Zhang W. (2024). Paraquat disrupts the blood–brain barrier by increasing IL-6 expression and oxidative stress through the activation of PI3K/AKT signaling pathway. Open Med..

[56] Berry C., La Vecchia C., Nicotera P. (2010). Paraquat and Parkinson’s disease. Cell Death Differ..

[57] Niso-Santano M., Bravo-San Pedro J.M., Gómez-Sánchez R., Climent V., Soler G., Fuentes J.M., González-Polo R.A. (2011). ASK1 overexpression accelerates paraquat-induced autophagy via endoplasmic reticulum stress. Toxicol. Sci..

[58] Manning-Bog A.B., McCormack A.L., Li J., Uversky V.N., Fink A.L., Di Monte D.A. (2002). The herbicide paraquat causes up-regulation and aggregation of α-synuclein in mice: Paraquat and α-synuclein. J. Biol. Chem..

[59] Shimizu K., Matsubara K., Ohtaki K., Fujimaru S., Saito O., Shiono H. (2003). Paraquat induces long-lasting dopamine overflow through the excitotoxic pathway in the striatum of freely moving rats. Brain Res..

[60] Cristovao A.C., Choi D.-H., Baltazar G., Beal M.F., Kim Y.-S. (2009). The role of NADPH oxidase 1–derived reactive oxygen species in paraquat-mediated dopaminergic cell death. Antioxid. Redox Signal..

[61] Hou L., Liu J., Sun F., Huang R., Chang R., Ruan Z., Wang Y., Zhao J., Wang Q. (2023). Integrin Mac1 mediates paraquat and maneb-induced learning and memory impairments in mice through NADPH oxidase–NLRP3 inflammasome axis-dependent microglial activation. J. Neuroinflamm..

[62] Songin M., Ossowska K., Kuter K., Strosznajder J.B. (2011). Original articleAlteration of GSK-3β in the hippocampus and other brain structures after chronic paraquat administration in rats. Folia Neuropathol..

[63] Shimizu K., Matsubara K., Ohtaki K., Shiono H. (2003). Paraquat leads to dopaminergic neural vulnerability in organotypic midbrain culture. Neurosci. Res..

[64] Songin M., Strosznajder J.B., Fitał M., Kuter K., Kolasiewicz W., Nowak P., Ossowska K. (2011). Glycogen Synthase Kinase 3β and Its Phosphorylated Form (Y216) in the Paraquat-Induced Model of Parkinsonism. Neurotox. Res..

[65] Tanner Caroline M., Kamel F., Ross G.W., Hoppin Jane A., Goldman Samuel M., Korell M., Marras C., Bhudhikanok Grace S., Kasten M., Chade Anabel R. (2011). Rotenone, Paraquat, and Parkinson’s Disease. Environ. Health Perspect..

[66] Pezzoli G., Cereda E. (2013). Exposure to pesticides or solvents and risk of Parkinson disease. Neurology.

[67] Tangamornsuksan W., Lohitnavy O., Sruamsiri R., Chaiyakunapruk N., Norman Scholfield C., Reisfeld B., Lohitnavy M. (2019). Paraquat exposure and Parkinson’s disease: A systematic review and meta-analysis. Arch. Environ. Occup. Health.

[68] Vaccari C., El Dib R., Gomaa H., Lopes L.C., De Camargo J.L. (2019). Paraquat and Parkinson’s disease: A systematic review and meta-analysis of observational studies. J. Toxicol. Environ. Health Part B.

[69] Weed D.L. (2021). Does paraquat cause Parkinson’s disease? A review of reviews. NeuroToxicology.

[70] Paul K.C., Cockburn M., Gong Y., Bronstein J., Ritz B. (2024). Agricultural paraquat dichloride use and Parkinson’s disease in California’s Central Valley. Int. J. Epidemiol..

[71] Schönbrunn E., Eschenburg S., Shuttleworth W.A., Schloss J.V., Amrhein N., Evans J.N.S., Kabsch W. (2001). Interaction of the herbicide glyphosate with its target enzyme 5-enolpyruvylshikimate 3-phosphate synthase in atomic detail. Proc. Natl. Acad. Sci. USA.

[72] de Andréa M.M., Peres T.B., Luchini L.C., Bazarin S., Papini S., Matallo M.B., Savoy V.L.T. (2003). Influence of repeated applications of glyphosate on its persistence and soil bioactivity. Pesqui. Agropecu. Bras..

[73] Baer K.N., Wexler P. (2005). Glyphosate. Encyclopedia of Toxicology.

[74] Henderson A.M., Gervais J.A., Luukinen B., Buhl K., Stone D., Cross A., Jenkins J., New Product Introduction (NPI) Center (2010). Glyphosate General Fact Sheet.

[75] Martinez A., Al-Ahmad A.J. (2019). Effects of glyphosate and aminomethylphosphonic acid on an isogeneic model of the human blood-brain barrier. Toxicol. Lett..

[76] Xu J., Li G., Wang Z., Si L., He S., Cai J., Huang J., Donovan M.D. (2016). The role of L-type amino acid transporters in the uptake of glyphosate across mammalian epithelial tissues. Chemosphere.

[77] Winstone J.K., Pathak K.V., Winslow W., Piras I.S., White J., Sharma R., Huentelman M.J., Pirrotte P., Velazquez R. (2022). Glyphosate infiltrates the brain and increases pro-inflammatory cytokine TNFα: Implications for neurodegenerative disorders. J. Neuroinflamm..

[78] Butterfield D.A., Pocernich C.B. (2003). The glutamatergic system and Alzheimer’s disease: Therapeutic implications. CNS Drugs.

[79] Kamat P.K., Tota S., Saxena G., Shukla R., Nath C. (2010). Okadaic acid (ICV) induced memory impairment in rats: A suitable experimental model to test anti-dementia activity. Brain Res..

[80] Cattani D., Cesconetto P.A., Tavares M.K., Parisotto E.B., De Oliveira P.A., Rieg C.E.H., Leite M.C., Prediger R.D.S., Wendt N.C., Razzera G. (2017). Developmental exposure to glyphosate-based herbicide and depressive-like behavior in adult offspring: Implication of glutamate excitotoxicity and oxidative stress. Toxicology.

[81] Cattani D., de Liz Oliveira Cavalli V.L., Rieg C.E.H., Domingues J.T., Dal-Cim T., Tasca C.I., Silva F.R.M.B., Zamoner A. (2014). Mechanisms underlying the neurotoxicity induced by glyphosate-based herbicide in immature rat hippocampus: Involvement of glutamate excitotoxicity. Toxicology.

[82] Limberger C., Ferreira P.C., Fontella F.U., Oliveira A.C.L.J., Salles G.B., Souza D.O., Zimmer E.R., de Souza D.G. (2020). Glyphosate-based herbicide alters brain amino acid metabolism without affecting blood-brain barrier integrity: Molecular and cell biology/others. Alzheimers Dement..

[83] Bartholomew S.K., Winslow W., Sharma R., Pathak K.V., Tallino S., Judd J.M., Leon H., Turk J., Pirrotte P., Velazquez R. (2024). Glyphosate exposure exacerbates neuroinflammation and Alzheimer’s disease-like pathology despite a 6-month recovery period in mice. J. Neuroinflamm..

[84] Hsiao C.C., Yang A.-M., Wang C., Lin C.-Y. (2023). Association between glyphosate exposure and cognitive function, depression, and neurological diseases in a representative sample of US adults: NHANES 2013–2014 analysis. Environ. Res..

[85] Ren J., Yu Y., Wang Y., Dong Y., Shen X., Eiser A. (2024). Association Between Urinary Glyphosate Exposure and Cognitive Impairment in Older Adults from NHANES 2013–2014. J. Alzheimers Dis..

[86] Yang T., Zhou L., Jing P., Bao Y., Gu L., Chen Y., Shi X., Wang H., Wang L., Wang S. (2025). Association of glyphosate exposure with frailty and all-cause mortality in general adults: A population-based cohort study. Ecotoxicol. Environ. Saf..

[87] Pu Y., Chang L., Qu Y., Wang S., Tan Y., Wang X., Zhang J., Hashimoto K. (2020). Glyphosate exposure exacerbates the dopaminergic neurotoxicity in the mouse brain after repeated administration of MPTP. Neurosci. Lett..

[88] Costas-Ferreira C., Durán R., Faro L.R.F. (2022). Toxic Effects of Glyphosate on the Nervous System: A Systematic Review. Int. J. Mol. Sci..

[89] Bloem B.R., Boonstra T.A. (2023). The inadequacy of current pesticide regulations for protecting brain health: The case of glyphosate and Parkinson’s disease. Lancet Planet. Health.

[90] Gui Y.-x., Fan X.-n., Wang H.-m., Wang G., Chen S.-d. (2012). Glyphosate induced cell death through apoptotic and autophagic mechanisms. Neurotoxicol. Teratol..

[91] Zheng Q., Yin J., Zhu L., Jiao L., Xu Z. (2018). Reversible Parkinsonism induced by acute exposure glyphosate. Park. Relat. Disord..

[92] Eriguchi M., Iida K., Ikeda S., Osoegawa M., Nishioka K., Hattori N., Nagayama H., Hara H. (2019). Parkinsonism Relating to Intoxication with Glyphosate. Intern. Med..

[93] Ojelade B.S., Durowoju O.S., Adesoye P.O., Gibb S.W., Ekosse G.-I. (2022). Review of Glyphosate-Based Herbicide and Aminomethylphosphonic Acid (AMPA): Environmental and Health Impacts. Appl. Sci..

[94] Dong Z., Han H., Li H., Bai Y., Wang W., Tu M., Peng Y., Zhou L., He W., Wu X. (2015). Long-term potentiation decay and memory loss are mediated by AMPAR endocytosis. J. Clin. Investig..

[95] Costa C., Sgobio C., Siliquini S., Tozzi A., Tantucci M., Ghiglieri V., Di Filippo M., Pendolino V., de Iure A., Marti M. (2012). Mechanisms underlying the impairment of hippocampal long-term potentiation and memory in experimental Parkinson’s disease. Brain.

[96] Izumi Y., O’Dell K.A., Zorumski C.F. (2023). The herbicide glyphosate inhibits hippocampal long-term potentiation and learning through activation of pro-inflammatory signaling. Res. Sq..

[97] da Silva K.N., Garbin Cappellaro L., Ueda C.N., Rodrigues L., Pertile Remor A., de Paula Martins R., Latini A., Glaser V. (2020). Glyphosate-based herbicide impairs energy metabolism and increases autophagy in C6 astroglioma cell line. J. Toxicol. Environ. Health Part A.

[98] Duque-Díaz E., Giraldo H.H., Rocha-Muñoz L.P., Coveñas R. (2022). Glyphosate, AMPA and glyphosate-based herbicide exposure leads to GFAP, PCNA and caspase-3 increased immunoreactive area on male offspring rat hypothalamus. Eur. J. Histochem. EJH.

[99] Izumi Y., O’Dell K.A., Zorumski C.F. (2024). Glyphosate as a direct or indirect activator of pro-inflammatory signaling and cognitive impairment. Neural Regen. Res..

[100] Tulchinsky T.H., Varavikova E.A., Tulchinsky T.H., Varavikova E.A. (2014). Chapter 9—Environmental and Occupational Health. The New Public Health.

[101] Kang H.K., Corn M. (1993). Dioxin, Health Effects. Handbook of Hazardous Materials.

[102] Nhung N.T.H., Nguyen X.T., Long V.D., Wei Y., Fujita T. (2022). A Review of Soil Contaminated with Dioxins and Biodegradation Technologies: Current Status and Future Prospects. Toxics.

[103] Committee to Review the Health Effects in Vietnam Veterans of Exposure to Herbicides (1994). Veterans and Agent Orange: Health Effects of Herbicides Used in Vietnam.

[104] Le Le T.H. (2018). Human health risk assessment of Agent orange/dioxin from contaminated soil in A Luoi district in central Vietnam. J. Vietnam Environ..

[105] Kim M.J., Marchand P., Henegar C., Antignac J.P., Alili R., Poitou C., Bouillot J.L., Basdevant A., Le Bizec B., Barouki R. (2011). Fate and complex pathogenic effects of dioxins and polychlorinated biphenyls in obese subjects before and after drastic weight loss. Environ. Health Perspect..

[106] Yi S.-W., Hong J.-S., Ohrr H., Yi J.-J. (2014). Agent Orange exposure and disease prevalence in Korean Vietnam veterans: The Korean veterans health study. Environ. Res..

[107] Medicine I.O. (2009). Veterans and Agent Orange: Update 2008.

[108] Lee W., Lee S., Kang S.K., Choi W.-J. (2024). Risk of Dementia in Korean Vietnam War Veterans. J. Prev. Alzheimers Dis..

[109] Martinez S., Yaffe K., Li Y., Byers A.L., Peltz C.B., Barnes D.E. (2021). Agent Orange Exposure and Dementia Diagnosis in US Veterans of the Vietnam Era. JAMA Neurol..

[110] Lee H.A., Kyeong S., Kim D.H. (2022). Long-term effects of defoliant exposure on brain atrophy progression in humans. Neurotoxicology.

[111] Yang Y., Cheon M., Kwak Y.T. (2016). Is Parkinson’s Disease with History of Agent Orange Exposure Different from Idiopathic Parkinson’s Disease?. Dement. Neurocogn. Disord..

[112] Lowes S., Sykes D., Breen C.M., Ragone L.J., Miller D.S. (2005). Multiple Components of 2,4-Dichlorophenoxyacetic Acid Uptake by Rat Choroid Plexus. J. Pharmacol. Exp. Ther..

[113] Institute of Medicine Committee to Review the Health Effects in Vietnam Veterans of Exposure to Herbicides (1996). Veterans and Agent Orange: Update 1996.

[114] Kim C.S., Pritchard J.B. (1993). Transport of 2,4,5-trichlorophenoxyacetic acid across the blood-cerebrospinal fluid barrier of the rabbit. J. Pharmacol. Exp. Ther..

[115] Kim C.S., Keizer R.F., Pritchard J.B. (1988). 2,4-Dichlorophenoxyacetic acid intoxication increases its accumulation within the brain. Brain Res..

[116] Sharifi Pasandi M., Hosseini Shirazi F., Gholami M.R., Salehi H., Najafzadeh N., Mazani M., Ghasemi Hamidabadi H., Niapour A. (2017). Epi/perineural and Schwann cells as well as perineural sheath integrity are affected following 2,4-D exposure. Neurotox. Res..

[117] de la Monte S.M., Goel A., Tong M., Delikkaya B. (2023). Agent Orange Causes Metabolic Dysfunction and Molecular Pathology Reminiscent of Alzheimer’s Disease. J. Alzheimers Dis. Rep..

[118] Xie H.Q., Ma Y., Fu H., Xu T., Luo Y., Liu Y., Chen Y., Xu L., Xia Y., Zhao B. (2021). New perspective on the regulation of acetylcholinesterase via the aryl hydrocarbon receptor. J. Neurochem..

[119] de la Monte S.M., Tong M. (2024). Agent Orange Herbicidal Toxin-Initiation of Alzheimer-Type Neurodegeneration. J. Alzheimers Dis..

[120] Yang Y., Giau V.V., An S.S.A., Kim S. (2018). Plasma Oligomeric Beta Amyloid in Alzheimer’s Disease with History of Agent Orange Exposure. Dement. Neurocogn. Disord..

[121] Williamson M.A., Gasiewicz T.A., Opanashuk L.A. (2004). Aryl Hydrocarbon Receptor Expression and Activity in Cerebellar Granule Neuroblasts: Implications for Development and Dioxin Neurotoxicity. Toxicol. Sci..

[122] Sánchez-Martín F.J., Fernández-Salguero P.M., Merino J.M. (2011). Aryl hydrocarbon receptor-dependent induction of apoptosis by 2, 3, 7, 8-tetrachlorodibenzo-p-dioxin in cerebellar granule cells from mouse. J. Neurochem..

[123] Furue M., Ishii Y., Tsukimori K., Tsuji G. (2021). Aryl Hydrocarbon Receptor and Dioxin-Related Health Hazards—Lessons from Yusho. Int. J. Mol. Sci..

[124] Xie H.Q., Xu H.M., Fu H.L., Hu Q., Tian W.J., Pei X.H., Zhao B. (2013). AhR-mediated effects of dioxin on neuronal acetylcholinesterase expression in vitro. Environ. Health Perspect..

[125] Abudahab S., Price E.T., Dozmorov M.G., Deshpande L.S., McClay J.L. (2023). The Aryl Hydrocarbon Receptor, Epigenetics and the Aging Process. J. Nutr. Health Aging.

[126] Thao P.N., Nishijo M., Tai P.T., Nghi T.N., Yokawa T., Hoa V.T., Tien T.V., Kien N.X., Anh T.H., Nishino Y. (2024). Impacts of dioxin exposure on brain connectivity estimated by DTI analysis of MRI images in men residing in contaminated areas of Vietnam. Front. Neurosci..

[127] Pandics T., Major D., Fazekas-Pongor V., Szarvas Z., Peterfi A., Mukli P., Gulej R., Ungvari A., Fekete M., Tompa A. (2023). Exposome and unhealthy aging: Environmental drivers from air pollution to occupational exposures. Geroscience.

[128] Song S., Kim J.Y., Lee Y., Jeong H., Kim S., Lee E.E. (2023). Effects of defoliant exposure and medication use on the development of Parkinson’s disease in veterans. Age Ageing.

[129] Bortolozzi A.A., Evangelista de Duffard A.M., Duffard R.O., Antonelli M.C. (2004). Effects of 2,4-dichlorophenoxyacetic acid exposure on dopamine D2-like receptors in rat brain. Neurotoxicol. Teratol..

[130] Buddhala C., Loftin S.K., Kuley B.M., Cairns N.J., Campbell M.C., Perlmutter J.S., Kotzbauer P.T. (2015). Dopaminergic, serotonergic, and noradrenergic deficits in Parkinson disease. Ann. Clin. Transl. Neurol..

[131] Russ T., Enders L., Zbiegly J.M., Potru P.S., Wurm J., Spittau B. (2023). 2,4-Dichlorophenoxyacetic Acid Induces Degeneration of mDA Neurons In Vitro. Biomedicines.

[132] Bortolozzi A.A., Duffard R., Evangelista de Duffard A.M. (2003). Asymmetrical development of the monoamine systems in 2, 4-dichlorophenoxyacetic acid treated rats. Neurotoxicology.

[133] Bortolozzi A., Duffard R., Antonelli M., Evangelista de Duffard A.M. (2002). Increased sensitivity in dopamine D(2)-like brain receptors from 2,4-dichlorophenoxyacetic acid (2,4-D)-exposed and amphetamine-challenged rats. Ann. N. Y. Acad. Sci..

[134] Ueda R.M.R., de Souza V.M., Magalhães L.R., Giuffrida R., Nai G.A. (2021). Alteration of object recognition memory after chronic exposure to dichlorophenoxyacetic acid (2, 4-D) in adult rats. Res. Soc. Dev..

[135] Oliveira G.H., Palermo-Neto J. (1993). Effects of 2,4-dichlorophenoxyacetic acid (2,4-D) on open-field behaviour and neurochemical parameters of rats. Pharmacol. Toxicol..

[136] de la Monte S.M., Goel A. (2022). Agent Orange reviewed: Potential role in peripheral neuropathy and neurodegeneration. J. Mil. Veterans’ Health.

[137] Rosso S.B., Di Paolo O.A., de Duffard A.M.E., Duffard R. (1997). Effects of 2,4-dichlorophenoxyacetic acid on central nervous system of developmental rats. Associated changes in ganglioside pattern. Brain Res..

[138] Shrestha S., Parks C.G., Umbach D.M., Richards-Barber M., Hofmann J.N., Chen H., Blair A., Beane Freeman L.E., Sandler D.P. (2020). Pesticide use and incident Parkinson’s disease in a cohort of farmers and their spouses. Environ. Res..

[139] Kamel F., Tanner C., Umbach D., Hoppin J., Alavanja M., Blair A., Comyns K., Goldman S., Korell M., Langston J. (2007). Pesticide exposure and self-reported Parkinson’s disease in the agricultural health study. Am. J. Epidemiol..

[140] Tanner C.M., Ross G.W., Jewell S.A., Hauser R.A., Jankovic J., Factor S.A., Bressman S., Deligtisch A., Marras C., Lyons K.E. (2009). Occupation and Risk of Parkinsonism: A Multicenter Case-Control Study. Arch. Neurol..

[141] Shilpha J., Song J., Jeong B.R. (2023). Ammonium Phytotoxicity and Tolerance: An Insight into Ammonium Nutrition to Improve Crop Productivity. Agronomy.

[142] Padappayil R.P., Borger J. (2023). Ammonia toxicity. StatPearls [Internet].

[143] New Jersey Department of Health (2016). Ammonium Chloride: Hazardous Substance Fact Sheet.

[144] Ott P., Larsen F.S. (2004). Blood-brain barrier permeability to ammonia in liver failure: A critical reappraisal. Neurochem. Int..

[145] Lockwood A.H., Finn R.D., Campbell J.A., Richman T.B. (1980). Factors that affect the uptake of ammonia by the brain: The blood-brain pH gradient. Brain Res..

[146] Kleidonas D., Hilfiger L., Lenz M., Häussinger D., Vlachos A. (2024). Ammonium chloride reduces excitatory synaptic transmission onto CA1 pyramidal neurons of mouse organotypic slice cultures. Front. Cell Neurosci..

[147] Hertz L., Peng L., Song D. (2015). Ammonia, like K+, stimulates the Na+, K+, 2 Cl− cotransporter NKCC1 and the Na+, K+-ATPase and interacts with endogenous ouabain in astrocytes. Neurochem. Res..

[148] Adlimoghaddam A., Sabbir M.G., Albensi B.C. (2016). Ammonia as a Potential Neurotoxic Factor in Alzheimer’s Disease. Front. Mol. Neurosci..

[149] Rose C. (2006). Effect of ammonia on astrocytic glutamate uptake/release mechanisms. J. Neurochem..

[150] Rose C., Kresse W., Kettenmann H. (2005). Acute insult of ammonia leads to calcium-dependent glutamate release from cultured astrocytes, an effect of pH. J. Biol. Chem..

[151] Skowrońska M., Albrecht J. (2012). Alterations of blood brain barrier function in hyperammonemia: An overview. Neurotox. Res..

[152] Marini J.C., Broussard S.R. (2006). Hyperammonemia increases sensitivity to LPS. Mol. Genet. Metab..

[153] Jo D., Kim B.C., Cho K.A., Song J. (2021). The Cerebral Effect of Ammonia in Brain Aging: Blood–Brain Barrier Breakdown, Mitochondrial Dysfunction, and Neuroinflammation. J. Clin. Med..

[154] Cheon S.Y., Kim M.-Y., Kim J., Kim E.J., Kam E.H., Cho I., Koo B.-N., Kim S.Y. (2023). Hyperammonemia induces microglial NLRP3 inflammasome activation via mitochondrial oxidative stress in hepatic encephalopathy. Biomed. J..

[155] Lazarenko R.M., DelBove C.E., Strothman C.E., Zhang Q. (2017). Ammonium chloride alters neuronal excitability and synaptic vesicle release. Sci. Rep..

[156] Fan P., Lavoie J., Lé N.L., Szerb J.C., Butterworth R.F. (1990). Neurochemical and electrophysiological studies on the inhibitory effect of ammonium ions on synaptic transmission in slices of rat hippocampus: Evidence for a postsynaptic action. Neuroscience.

[157] Dagra A., Miller D.R., Lin M., Gopinath A., Shaerzadeh F., Harris S., Sorrentino Z.A., Støier J.F., Velasco S., Azar J. (2021). α-Synuclein-induced dysregulation of neuronal activity contributes to murine dopamine neuron vulnerability. npj Park. Dis..

[158] Venda L.L., Cragg S.J., Buchman V.L., Wade-Martins R. (2010). α-Synuclein and dopamine at the crossroads of Parkinson’s disease. Trends Neurosci..

[159] Fan P., Szerb J.C. (1993). Effects of ammonium ions on synaptic transmission and on responses to quisqualate and N-methyl-D-aspartate in hippocampal CA1 pyramidal neurons in vitro. Brain Res..

[160] Rama Rao K.V., Jayakumar A.R., Tong X., Alvarez V.M., Norenberg M.D. (2010). Marked potentiation of cell swelling by cytokines in ammonia-sensitized cultured astrocytes. J. Neuroinflamm..

[161] Rama Rao K.V., Jayakumar A.R., Norenberg M.D. (2005). Role of oxidative stress in the ammonia-induced mitochondrial permeability transition in cultured astrocytes. Neurochem. Int..

[162] Komatsu A., Iida I., Nasu Y., Ito G., Harada F., Kishikawa S., Moss S.J., Maeda T., Terunuma M. (2022). Ammonia induces amyloidogenesis in astrocytes by promoting amyloid precursor protein translocation into the endoplasmic reticulum. J. Biol. Chem..

[163] Busche M.A., Eichhoff G., Adelsberger H., Abramowski D., Wiederhold K.-H., Haass C., Staufenbiel M., Konnerth A., Garaschuk O. (2008). Clusters of hyperactive neurons near amyloid plaques in a mouse model of Alzheimer’s disease. Science.

[164] Ismail F.S., Faustmann T.J., Corvace F., Tsvetanova A., Moinfar Z., Faustmann P.M. (2021). Ammonia induced microglia activation was associated with limited effects on connexin 43 and aquaporin 4 expression in an astrocyte-microglia co-culture model. BMC Neurosci..

[165] Yu C., Huang X., Xu Y., Li H., Su J., Zhong J., Kang J., Liu Y., Sun L. (2013). Lysosome dysfunction enhances oxidative stress-induced apoptosis through ubiquitinated protein accumulation in Hela cells. Anat. Rec. Adv. Integr. Anat. Evol. Biol..

[166] Sala G., Arosio A., Stefanoni G., Melchionda L., Riva C., Marinig D., Brighina L., Ferrarese C. (2013). Rotenone upregulates alpha-synuclein and myocyte enhancer factor 2D independently from lysosomal degradation inhibition. BioMed Res. Int..

[167] Pidoplichko V.I., Dani J.A. (2006). Acid-sensitive ionic channels in midbrain dopamine neurons are sensitive to ammonium, which may contribute to hyperammonemia damage. Proc. Natl. Acad. Sci. USA.

[168] Yue T., Li B., Gu L., Huang J., Verkhratsky A., Peng L. (2019). Ammonium induced dysfunction of 5-HT2B receptor in astrocytes. Neurochem. Int..

[169] Kang M.J., Gil S.J., Koh H.C. (2009). Paraquat induces alternation of the dopamine catabolic pathways and glutathione levels in the substantia nigra of mice. Toxicol. Lett..

[170] Ramachandiran S., Hansen J.M., Jones D.P., Richardson J.R., Miller G.W. (2007). Divergent mechanisms of paraquat, MPP+, and rotenone toxicity: Oxidation of thioredoxin and caspase-3 activation. Toxicol. Sci..

[171] Murguiondo-Pérez R., Vidal Alcántar-Garibay Ó., Weintraub Ben-Zión E., Blancarte-Hernández E., Tejerina-Marion E., Cristina Loza-López E., Galindo-Ruiz M., Rivera-Monroy G. (2022). The influence of gut brain axis in neurodegenerative diseases: Short review. Rev. Mex. Neurocienc..

[172] Lehman P.C., Cady N., Ghimire S., Shahi S.K., Shrode R.L., Lehmler H.-J., Mangalam A.K. (2023). Low-dose glyphosate exposure alters gut microbiota composition and modulates gut homeostasis. Environ. Toxicol. Pharmacol..

[173] Vogt N.M., Kerby R.L., Dill-McFarland K.A., Harding S.J., Merluzzi A.P., Johnson S.C., Carlsson C.M., Asthana S., Zetterberg H., Blennow K. (2017). Gut microbiome alterations in Alzheimer’s disease. Sci. Rep..

[174] Shinabarger D., Braymer H. (1986). Glyphosate catabolism by Pseudomonas sp. strain PG2982. J. Bacteriol..

[175] Nie C.L., Wang X.S., Liu Y., Perrett S., He R.Q. (2007). Amyloid-like aggregates of neuronal tau induced by formaldehyde promote apoptosis of neuronal cells. BMC Neurosci..

[176] Vitku J., Hill M., Kolatorova L., Kubala Havrdova E., Kancheva R. (2022). Steroid Sulfation in Neurodegenerative Diseases. Front. Mol. Biosci..

[177] Heafield M.T., Fearn S., Steventon G.B., Waring R.H., Williams A.C., Sturman S.G. (1990). Plasma cysteine and sulphate levels in patients with motor neurone, Parkinson’s and Alzheimer’s disease. Neurosci. Lett..

[178] Samsel A., Seneff S. (2013). Glyphosate’s Suppression of Cytochrome P450 Enzymes and Amino Acid Biosynthesis by the Gut Microbiome: Pathways to Modern Diseases. Entropy.

[179] Wang K., Zhang C., Zhang B., Li G., Shi G., Cai Q., Huang M. (2022). Gut dysfunction may be the source of pathological aggregation of alpha-synuclein in the central nervous system through Paraquat exposure in mice. Ecotoxicol. Environ. Saf..

[180] Tu P., Gao B., Chi L., Lai Y., Bian X., Ru H., Lu K. (2019). Subchronic low-dose 2, 4-D exposure changed plasma acylcarnitine levels and induced gut microbiome perturbations in mice. Sci. Rep..

[181] Petriello M.C., Hoffman J.B., Vsevolozhskaya O., Morris A.J., Hennig B. (2018). Dioxin-like PCB 126 increases intestinal inflammation and disrupts gut microbiota and metabolic homeostasis. Environ. Pollut..

[182] Chen L. (2020). Visual system: An understudied target of aquatic toxicology. Aquat. Toxicol..

[183] Li Y., Pan L., Zeng X., Zhang R., Li X., Li J., Xing H., Bao J. (2021). Ammonia exposure causes the imbalance of the gut-brain axis by altering gene networks associated with oxidative metabolism, inflammation and apoptosis. Ecotoxicol. Environ. Saf..

[184] Ajasa A. (2025). 70 countries have banned this pesticide. It’s still for sale in the U.S. The Washington Post.

[185] Gewin V. (2025). Is There Enough Evidence of Health Risks for the EPA to Ban Paraquat?.

[186] Wadman M. (2025). EPA will soon weigh in on weed killer that may cause Parkinson’s disease. Science.

[187] Williams G.M., Kroes R., Munro I.C. (2000). Safety evaluation and risk assessment of the herbicide Roundup and its active ingredient, glyphosate, for humans. Regul. Toxicol. Pharmacol..

[188] IARC (2017). Evaluation of Carcinogenic Risks to Humans.

[189] EPA (2022). Withdrawal of the Glyphosate Interim Registration Review Decision.

[190] Erickson B.E. (2021). Bayer to End Glyphosate Sales to US Consumers.

[191] Rice M. (2025). Glyphosate Ban. https://www.motleyrice.com/news/glysophate-ban.

[192] EFSA (2023). Glyphosate: No Critical Areas of Concern; Data Gaps Identified. https://www.efsa.europa.eu/en/news/glyphosate-no-critical-areas-concern-data-gaps-identified.

[193] European Commission (2023). Glyphosate: Commission Adopts a Renewal for 10 Years.

[194] Hammond L.E. The Regulatory History of 2,4-D in the United States. 2015 Benefits and Economic Assessment of 2,4-D and The Phenoxy Herbicides in the US. https://24d.info/wp-content/uploads/2020/08/2_Welfare_Analysis_of_Prohibition_of_24-D_03-10-2018.pdf.

[195] Environmental Protection Agency (2017). 2,4-D; Pesticide Tolerances.

[196] EPA (2023). Ammonium Chloride. https://cdxapps.epa.gov/oms-substance-registry-services/substance-details/179416.

[197] OSHA (2024). Occupational Safety and Health Administration.

